# Hydrogen Compatibility of Commercial Silicone Elastomer Seals: Multiscale Mechanical and Surface Characterization Following High-Pressure Hydrogen Exposure

**DOI:** 10.3390/polym18212733

**Published:** 2026-11-03

**Authors:** Nitesh Subedi, Alfredo Becerril Corral, Md. Ariful Islam, Md. Monjur Hossain Bhuiyan, Zahed Siddique

**Affiliations:** School of Aerospace and Mechanical Engineering, University of Oklahoma, Norman, OK 73019, USA

**Keywords:** hydrogen compatibility, silicone elastomer, high-pressure hydrogen, elastomeric seals, mechanical characterization, quantitative SEM image analysis

## Abstract

Commercial silicone O-rings were exposed to hydrogen pressures up to 7000 psi for 192 h and evaluated using mechanical, data-driven, and microstructural characterization. ASTM D1414-based O-ring tensile testing was complemented by multivariate and functional analyses, while surface evolution was quantified using SEM-based image analysis and spatial statistics. Inclusion of an unexposed 0 psi mechanical control revealed a non-monotonic exposure-associated response. Several hydrogen-exposed groups exhibited greater ultimate elongation, tensile toughness, and structural loading work than the unexposed control, whereas these quantities decreased at 7000 psi relative to selected lower-pressure exposed groups. Welch ANOVA identified significant overall group effects for tensile strength, ultimate elongation, toughness, and structural loading work. The normalized tensile-curve shapes and several stiffness-related descriptors showed smaller relative differences than the failure-related properties. These results demonstrate that hydrogen exposure was associated with changes in deformability and failure response, but the tensile data alone do not establish the molecular mechanism responsible for these changes. Quantitative SEM analysis revealed pressure-associated differences in localized morphology and spatial organization, including a tendency toward reduced dark-feature number density and variation in the magnitude and length scale of feature clustering among the analyzed conditions, without evidence of catastrophic surface degradation in the examined SEM fields. Overall, the investigated silicone exhibited non-monotonic exposure-associated changes in mechanical response together with localized pressure-associated surface evolution, without evidence of catastrophic surface degradation in the analyzed SEM fields. These findings provide comparative post-exposure material-response data for commercial silicone O-rings under high-pressure hydrogen conditions.

## 1. Introduction

### 1.1. Background and Motivation

Hydrogen is expected to play a critical role in the transition toward sustainable energy systems by enabling low-carbon pathways for transportation, stationary power generation, and industrial decarbonization [1,2,3]. Its extremely low molecular weight and low volumetric energy density, however, require storage and transportation under high-pressure conditions, placing stringent demands on materials used throughout hydrogen infrastructure [2,4,5]. Elastomeric seals are essential components in pressure vessels, compressors, valves, dispensing systems, piping connections, and fuel-cell balance-of-plant equipment, where they maintain leak-tight interfaces under varying mechanical and environmental conditions [6,7].

The reliability of hydrogen systems depends strongly on the ability of these seals to preserve mechanical integrity during prolonged exposure to pressurized hydrogen [6,7]. Elastomers maintain sealing through large elastic deformation and intimate contact with mating surfaces [7,8]. Hydrogen-induced changes in mechanical behavior may reduce sealing contact pressure, alter deformation characteristics, and compromise sealing effectiveness, thereby increasing leakage risk and reducing operational reliability [1,8,9,10].

Hydrogen exposure can alter elastomer performance through coupled diffusion, sorption, swelling, and desorption processes [4,6,9]. Hydrogen uptake may modify polymer-chain mobility and deformation behavior, while depressurization can generate internal gas-pressure gradients that promote localized stress concentrations, microvoid formation, blistering, and crack initiation [1,8,9,10]. The severity of these effects depends on polymer chemistry, crosslink density, filler morphology, free-volume distribution, hydrogen permeability, and desorption kinetics [4,7,8,9,11]. Consequently, elastomers with similar nominal properties may respond differently under hydrogen exposure, emphasizing the need for material-specific evaluation under relevant service conditions [1,10].

Silicone rubber is particularly relevant because of its broad thermal stability, flexibility, environmental resistance, and use in hydrogen-related sensors, valves, connectors, and auxiliary sealing components. Its siloxane backbone provides high chain flexibility and comparatively large free volume, which generally result in higher gas permeability than many conventional sealing elastomers [12]. Hydrogen transport and mechanical evolution in silicone may therefore differ substantially from those of elastomers such as EPDM or fluorocarbon rubber, motivating dedicated investigation [1,10].

The mechanical response of silicone seals is especially important because tensile resistance, deformation capacity, and stiffness influence seal contact pressure, load transfer, sealing force, and resistance to mechanical failure [6,8,10]. Under repeated loading, pressure fluctuations, or decompression events, changes in these properties may reduce sealing reliability even without visible macroscopic damage [1,6,10,12]. Understanding how prolonged hydrogen exposure affects commercial silicone O-rings is therefore important for material selection, component design, and reliability assessment in hydrogen infrastructure [4,6,11].

### 1.2. Current Understanding of Hydrogen–Elastomer Interactions

High-pressure hydrogen can influence elastomer performance through coupled diffusion, sorption, swelling, and desorption processes that alter mechanical behavior during and after exposure [7,9]. Hydrogen uptake may temporarily modify stiffness, chain mobility, and deformation characteristics, whereas subsequent depressurization can generate internal gas-pressure gradients that promote localized damage such as microvoids, blistering, interfacial debonding, and surface cracking [1,5,6]. Consequently, post-exposure mechanical behavior may reflect contributions from both reversible hydrogen–polymer interactions and irreversible microstructural changes rather than a single degradation mechanism [9,10].

The measured response also depends strongly on exposure and testing conditions. Pressure, aging duration, depressurization rate, specimen geometry, degassing period, and mechanical testing protocol can substantially influence the observed post-exposure properties [6,7]. Partial recovery of some mechanical properties after hydrogen desorption has been reported, whereas residual deformation, compression set, and internal or surface damage may persist [7,8]. Therefore, hydrogen compatibility should be evaluated in the context of the complete exposure and testing history rather than pressure alone [4,9].

Formulation-controlled studies further demonstrate that hydrogen response can depend strongly on filler type and polymer–filler interactions. Yamabe and Nishimura [13] investigated sulfur-vulcanized NBR prepared as unfilled, carbon-black-filled, and silica-filled formulations following exposure to hydrogen pressures up to 100 MPa. The volume increase remained relatively small at lower pressures but increased at pressures above approximately 10 MPa, while the tensile elastic modulus decreased with increasing swelling. The filled formulations exhibited smaller volume increases than the unfilled material, and the fracture response of the filled compounds was influenced by changes associated with the filler–rubber interface. Related investigations of formulation-controlled EPDM and NBR systems also demonstrated that carbon black and silica can differently affect hydrogen uptake and blister formation [14]. Under cyclic high-pressure hydrogen exposure, silica-filled NBR was subsequently reported to exhibit a reduction in rubbery storage modulus together with changes in the filler–gel structure, whereas comparable changes were less pronounced in the unfilled and carbon-black-filled formulations [15].

These studies demonstrate that filler chemistry, filler–polymer interactions, and formulation can substantially influence the mechanical and morphological response of elastomers to high-pressure hydrogen. Direct quantitative transfer of these observations to the present silicone material is not appropriate because the polymer chemistry, formulation, exposure history, and test conditions differ. In particular, the present work examines a commercially formulated Parker S1138 silicone O-ring whose proprietary filler and additive composition is not disclosed. Nevertheless, the formulation-controlled literature provides an important basis for interpreting the present results cautiously and reinforces the need to distinguish measured macroscopic mechanical changes from unverified filler- or molecular-scale mechanisms.

Increasing attention has also been directed toward complete sealing geometries rather than simplified laboratory specimens. Although conventional dumbbell specimens are useful for determining intrinsic material properties, they do not fully reproduce the geometry, stress distribution, and manufacturing variability of commercial seals [6,10]. Testing complete O-rings therefore provides a more engineering-relevant basis for relating hydrogen exposure to changes in deformation and mechanical durability.

Despite these advances, integrated studies of commercial silicone O-rings remain limited. Previous hydrogen-elastomer research has largely emphasized permeability, swelling, compression set, rapid gas decompression resistance, or qualitative damage observations [6,9]. Image-based characterization methods, including deep-learning-assisted damage detection, have also been investigated for hydrogen-exposed NBR, demonstrating the potential of computational approaches for identifying localized morphological damage [16]. Fewer studies have combined pressure-dependent tensile properties, stiffness evolution, energy absorption, statistical variability, short-term stress relaxation, and quantitative surface characterization to determine which aspects of silicone-seal performance are most sensitive to hydrogen exposure [10,11]. This distinction is important because the preservation of stiffness or the overall deformation pathway does not necessarily imply the preservation of strength, deformability, or energy-absorption capacity.

The contribution of the present study is not the application of additional statistical descriptors alone, but the identification of how different mechanical response regimes of a commercial silicone O-ring change relative to a directly tested unexposed reference following prolonged fixed-pressure hydrogen exposure. Previous silicone O-ring investigations have primarily emphasized blister formation, extrusion fracture, leakage, and sealing durability during pressurization–depressurization cycling. For example, filled silicone O-rings have been evaluated under 100 hydrogen pressure cycles at 70 MPa and 30 °C with emphasis on backup-ring configuration, extrusion damage, and sealing performance, while other studies have examined blister development in VMQ O-rings following exposure and decompression. Long-duration cyclic testing has also demonstrated silicone O-ring durability over thousands of pressure cycles under specific sealing configurations.

The present study addresses a different but complementary question by quantifying the post-exposure tensile response of commercial silicone O-rings after 192 h of fixed-pressure hydrogen conditioning over 500–7000 psi and comparing the exposed specimens directly with a same-batch unexposed control. The results show that the response is not characterized by uniform deterioration or improvement. Failure-related quantities, particularly ultimate elongation and loading work to failure, exhibit comparatively large and non-monotonic exposure-associated differences, whereas resistance to deformation at the common 40% elongation state remains within a much narrower range. Thus, a comparatively similar intermediate-deformation stiffness does not imply the preservation of terminal deformation or failure behavior.

This response also differs from that previously observed for other commercial elastomer chemistries investigated under prolonged high-pressure hydrogen exposure. For example, prior FKM testing under a comparable 192 h exposure duration and pressures up to 7000 psi showed a pronounced high-pressure increase in load-bearing response accompanied by reduced displacement capacity. In contrast, the present silicone specimens exhibit substantially increased deformation capacity at several exposed conditions and a reduction in terminal properties at 7000 psi relative to selected lower-pressure groups. These differences reinforce that hydrogen-exposed elastomer behavior is material specific and cannot be inferred directly from results obtained for EPDM, NBR, or FKM [7,9].

### 1.3. Research Objectives

The objective of this study is to determine whether prolonged high-pressure hydrogen exposure affects the failure resistance, deformation resistance, time-dependent mechanical response, and surface integrity of commercial silicone O-ring seals to the same extent, or whether specific response modes are preferentially affected. The work therefore quantifies the pressure-dependent evolution of tensile behavior, stiffness characteristics, energy absorption, short-term viscoelastic relaxation, and surface morphology following controlled hydrogen aging. Mechanical performance is evaluated through force–displacement and stress–strain responses together with characteristic tensile parameters, stiffness descriptors, and energy-based metrics. By comparing these complementary response categories, the study aims to identify the mechanical indicators that are most sensitive to hydrogen exposure and thereby provide an engineering basis for material screening and reliability assessment of silicone seals used in high-pressure hydrogen systems.

In addition to conventional mechanical characterization, this study employs statistical and data-driven analyses, including coefficient of variation, principal component analysis, Pearson correlation analysis, functional data analysis, functional principal component analysis, and stress–relaxation modeling, to evaluate the consistency, variability, and pressure-dependent evolution of the mechanical response. Hydrogen exposure pressures up to 7000 psi (≈48 MPa) were selected to represent conditions relevant to high-pressure hydrogen storage, compression, and distribution systems [2,4]. Unlike many previous investigations based on standard sheet or dumbbell specimens, the present work evaluates complete commercial O-ring seals using an ASTM D1414 [10]-based testing approach to better replicate practical sealing components used in engineering applications [6,10]. The resulting experimental framework provides insight into the mechanical durability of silicone seals and supports material selection and reliability assessment for future hydrogen infrastructure.

## 2. Materials and Experimental Methods

The commercial silicone elastomer investigated in this study and the experimental procedures adopted for hydrogen aging and post-exposure characterization are described in the following sections.

### 2.1. Silicone Elastomer

**Figure 1:**
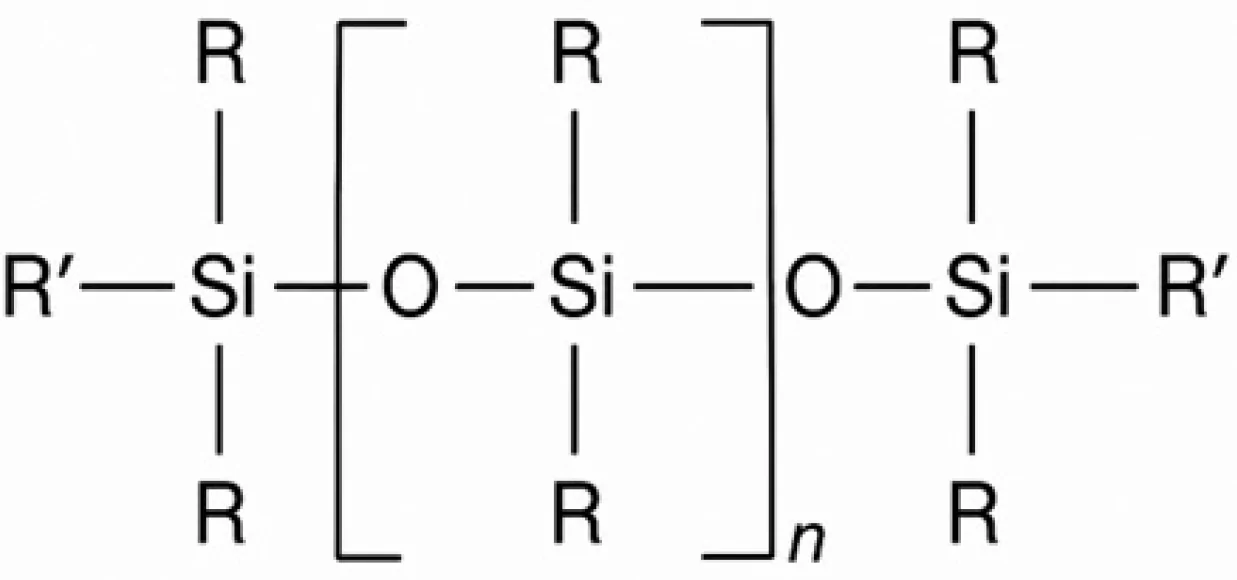
Generalized chemical structure of silicone (polysiloxane) elastomers showing the repeating siloxane backbone (Si-O-Si). The side groups R and terminal groups R′ represent organic substituents, most commonly methyl groups in commercial silicone formulations.

Commercial silicone rubber O-rings were selected because of their widespread use in sealing systems requiring flexibility over broad service-temperature ranges [12]. Silicone elastomers are based on a crosslinked polysiloxane (Si-O-Si) backbone with organic side groups, most commonly methyl groups, as illustrated schematically in Figure 1 [12]. The flexibility of the siloxane backbone contributes to the characteristic elasticity and thermal stability of silicone materials [12].

A distinguishing characteristic of silicone is its relatively high gas permeability compared with many engineering elastomers. Consequently, hydrogen absorption, diffusion, desorption, and the resulting mechanical response may differ from those of less permeable sealing materials such as FKM, NBR, and EPDM [1,9,10]. This behavior motivates a dedicated evaluation of commercial silicone seals under high-pressure hydrogen exposure [4,9].

### 2.2. Material Specifications

The investigated specimens were commercially manufactured high-temperature silicone O-rings obtained from McMaster-Carr (Elmhurst, IL, USA), supplier part number 9396K35. The product is identified by the supplier as Parker S1138 silicone rubber and was supplied as a finished industrial O-ring with AS568 [17] Dash-214 geometry.

The O-rings had a nominal inner diameter of 0.984 in (25.0 mm), outer diameter of 1.262 in (32.05 mm), and cross-sectional diameter of 0.139 in (3.53 mm). The nominal hardness was 70 Shore A. According to the supplier specifications, the product conforms to ASTM D2000 [18], SAE AS568 [17], and SAE J200 [19] and has a specified service-temperature range of −60 °F to 400 °F (approximately −51 °C to 204 °C). The commercial product is also listed as compliant with FDA 21 CFR 177.2600 and USP Class VI requirements.

The supplier provides the O-rings with a material certificate containing a traceable lot number, and the cure date is identified on the product packaging. All specimens used in the present mechanical comparison, including the unexposed 0 psi control specimens, were obtained from the same manufacturing batch and lot.

The exact proprietary formulation of Parker S1138, including the curing chemistry, filler identity and loading, additive package, and crosslink density, was not disclosed in the available supplier information. These compositional quantities are therefore not assumed or inferred in the present study.

The principal material specifications and dimensions of the commercial silicone O-rings investigated in this study are summarized in Table 1.

### 2.3. Significance of Commercial Silicone Materials

The exact formulation of the investigated silicone elastomer is proprietary and was not disclosed by the manufacturer. Commercial silicone sealing compounds generally contain reinforcing fillers, crosslinking agents, processing aids, and stabilizing additives that are optimized to achieve the desired balance between mechanical strength, elasticity, thermal stability, and environmental durability [12]. In filled elastomeric systems, the macroscopic mechanical response can also be influenced by filler–polymer interactions and deformation-induced rearrangement of the reinforcing network, which contribute to nonlinear deformation behavior [20]. Although formulation details such as filler loading, crosslink density, and curing chemistry can influence hydrogen transport and mechanical performance, these parameters were intentionally not varied in the present study [9,12].

Instead, this work evaluates commercially available sealing products that are representative of components currently used in industrial applications. This approach provides engineering-relevant information regarding the mechanical behavior of practical silicone seals following exposure to high-pressure hydrogen rather than examining the influence of individual formulation variables. Consequently, the results can support material screening and reliability assessment for hydrogen storage, compression, transportation, and dispensing applications [4].

### 2.4. Hydrogen Aging Protocol

Hydrogen aging was performed using the high-pressure exposure system and general procedure reported previously [10]. All silicone O-rings, including the unexposed controls, were obtained from the same manufacturing batch and lot. Exposed specimens were conditioned at 500, 1000, 2000, 5000, and 7000 psi for 192 h at approximately 25 °C under ambient laboratory conditions.

For each pressure condition, all replicate O-rings were exposed simultaneously in a single reactor run. The exposure sequence was 7000, 5000, 2000, 1000, and 500 psi, with each batch fully exposed, depressurized, and mechanically tested before the next pressure condition was initiated. No intermediate pressure cycling was used.

After 192 h, the reactor was depressurized at approximately 70 bar min^−1^ to atmospheric pressure. Because the same depressurization rate was used from different initial pressures, the total depressurization time varied among conditions. The reactor was then opened, specimens were removed and transferred to the tensile system, mounted on the fixture, and tested within approximately 15 min. This interval represented the practical time required for specimen retrieval, transfer, and mounting rather than an intentional degassing or recovery period.

**Figure 2:**
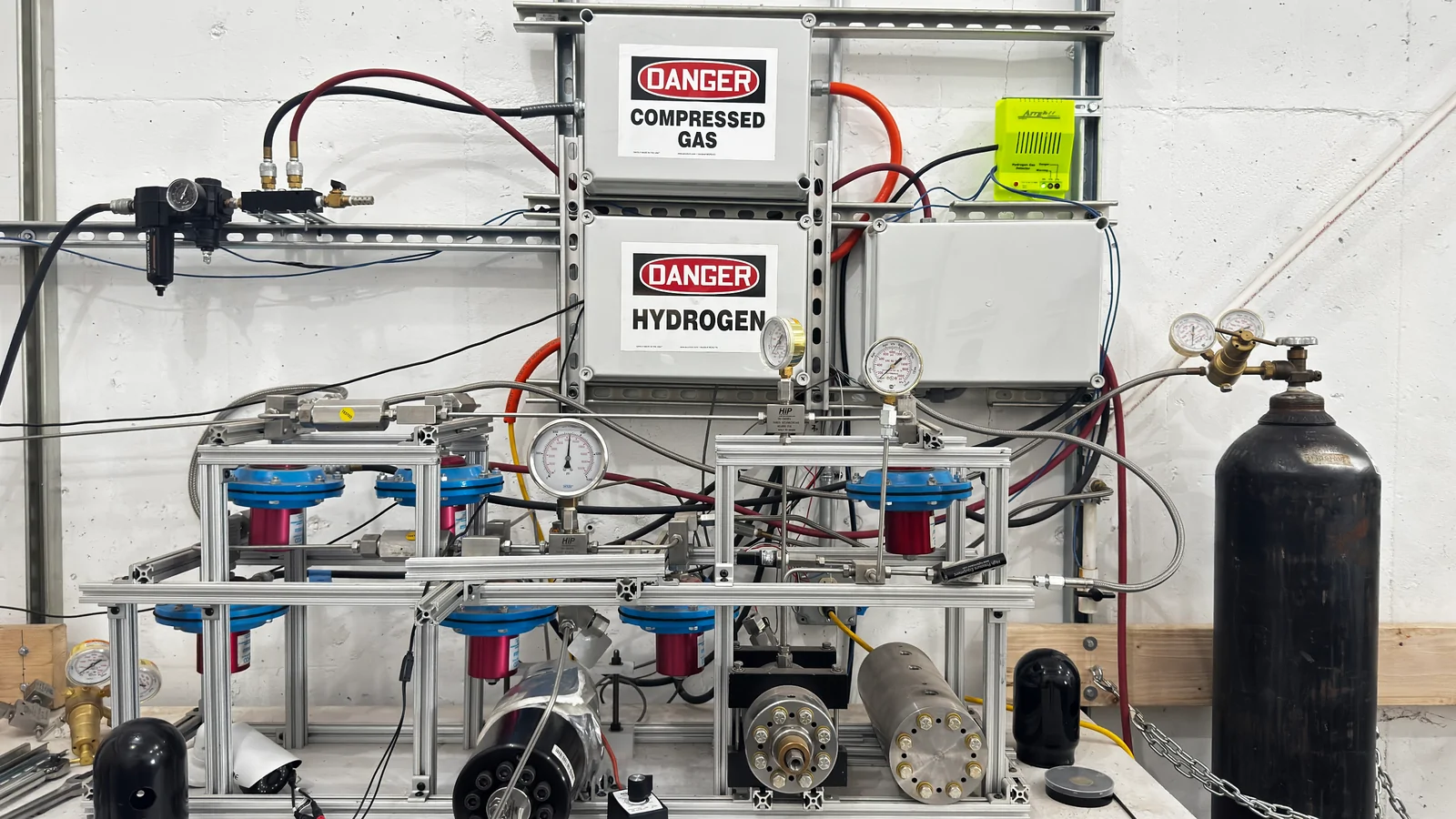
Experimental high-pressure hydrogen aging system used for the elastomer exposure experiments. The setup consists of a compressed hydrogen supply cylinder, pressure regulation and safety control components, hydrogen detection system, and a high-pressure aging reactor (GC-9; High Pressure Equipment Company, Erie, PA, USA) used to expose elastomer O-ring specimens to controlled hydrogen pressures [10].

The reported sample size at each pressure therefore represents individually tested O-ring specimens, while each pressure condition corresponded to one reactor exposure run. The high-pressure hydrogen aging system used for the exposure experiments is shown in Figure 2.

The tensile behavior of the silicone O-rings was evaluated directly using complete commercial O-ring specimens with the spool-type loading geometry and stress–elongation calculation framework described in ASTM D1414 [21]. The experimental configuration was identical for the unexposed and hydrogen-exposed specimens and followed the same general O-ring tensile methodology used in our previous study [10].

The O-ring was mounted between two cylindrical ball-bearing spools positioned sufficiently close to avoid initial pre-stretch. Displacement-controlled loading was performed using a machine-programmed velocity of 0.0200 in s^−1^, corresponding to approximately 0.508 mm s^−1^ (30.5 mm min^−1^). This loading rate was used consistently for all specimens as part of the combined tensile and intermediate stress–relaxation experimental protocol.

We note that this loading rate is lower than the 500 ± 50 mm min^−1^ crosshead speed specified for the standard ASTM D1414 [21] tensile procedure. Accordingly, the present testing procedure is described as ASTM D1414-based rather than fully conforming to the standard tensile-rate requirement. Because elastomeric tensile response can depend on loading rate, the absolute tensile properties reported here should not be interpreted as directly equivalent to values obtained using the standard ASTM D1414 crosshead speed. However, the identical loading rate, fixture configuration, specimen geometry, and data-processing procedure were applied to all unexposed and hydrogen-exposed specimens, providing a consistent basis for within-study comparison of exposure-associated mechanical differences.

Following ASTM D1414, the effective gauge length was determined from the instantaneous center-to-center spool spacing during loading. The stressed internal circumference of the O-ring was calculated as:

$$L=2D+\pi d_{s}$$

where D is the center-to-center distance between the two spools and $d_{s}\;$is the spool diameter. Engineering strain was determined from the change in the stressed circumference relative to the initial circumference, while engineering stress was calculated by normalizing the measured tensile force by twice the original cross-sectional area of one O-ring strand, accounting for the two load-bearing strands acting in parallel during spool-type tensile loading. Consequently, the tensile response was evaluated using both the conventional force–displacement curves prescribed by ASTM D1414 and the corresponding engineering stress–strain curves derived from the measured specimen geometry.

The complete force–displacement history was continuously recorded until specimen rupture. From these data, characteristic tensile parameters including peak tensile force, tensile strength, elongation at break, engineering stress, engineering strain, stiffness, and energy-based mechanical descriptors were subsequently determined as described in Section 2.7. For specimens subjected to short-term stress–relaxation testing, the tensile loading sequence included an intermediate constant-deformation hold before rupture. Loading was first applied to the prescribed hold deformation, the crosshead position was maintained constant during the relaxation period, and displacement-controlled tensile loading was subsequently resumed and continued until specimen rupture.

Consequently, the present methodology enables a direct comparative evaluation of commercially available sealing materials while maintaining consistent fixture geometry, loading rate, specimen geometry, and data-processing procedures across all conditions.

### 2.5. Data Processing and Mechanical Parameters

The raw tensile data were acquired as time-resolved force and displacement measurements from the universal testing machine. Data processing followed the general procedure reported in our previous study of hydrogen-aged EPDM and NBR O-rings [10], with additional calculations introduced to obtain engineering stress–strain responses and the mechanical descriptors used in the present study.

Prior to analysis, non-physical measurements associated with preload, actuator positioning, and post-fracture machine motion were removed. Negative initial force values were excluded, and each force–displacement record was truncated at specimen rupture. The same processing procedure was applied to all specimens. Following ASTM D1414, the stressed internal circumference of the O-ring during loading was determined from the instantaneous spool spacing:

$$L=2D+\pi d_{s}$$

where D is the center-to-center spool distance and $d_{s}\;$is the spool diameter.

The engineering strain was calculated as:

$$\varepsilon =\frac{L-L_{0}}{L_{0}}$$

where $L_{0}\;$is the initial stressed circumference and L is the instantaneous stressed circumference.

The engineering stress was determined from:

$$\sigma =\frac{F}{{2A}_{0}}$$

where F is the measured tensile force and $A_{0\;}$is the original cross-sectional area of the O-ring. The factor of 2 accounts for the two load-bearing strands acting in parallel during tensile loading. The maximum value of the resulting engineering stress–strain response was taken as the engineering tensile strength. The original cross-sectional area was calculated from:

$$A_{0}=\frac{\pi d^{2}}{4}$$

where d is the undeformed cross-sectional diameter of the O-ring.

The processed data were used to determine peak tensile force, tensile strength, engineering strain at failure, early-stage tangent modulus, structural stiffness, secant stiffness, tensile toughness, and structural loading work, as defined in Section 2.7.

To compare deformation pathways independently of absolute magnitude, the force, displacement, and time histories were normalized as:

$$F_{norm}=\frac{F}{F_{max}},$$

$$\delta_{norm}=\frac{\delta }{\delta_{max}},$$

$$t_{norm}=\frac{t}{t_{max}},$$

where $F_{max}$, $\delta_{max}$, and $t_{max}\;$represent the maximum force, maximum displacement, and time corresponding to the peak force, respectively. Normalization removes differences in absolute magnitude while preserving the characteristic shape of the tensile response, allowing a direct comparison of deformation evolution under different hydrogen pressures.

Independent O-ring specimens were mechanically evaluated at each condition, with sample sizes of *n* = 5, 3, 6, 4, 4, and 3 for 0, 500, 1000, 2000, 5000, and 7000 psi, respectively. A minimum of three specimens was evaluated at every condition, while additional same-lot specimens were available and tested at selected conditions, resulting in unequal replicate numbers across the experimental matrix. All valid mechanical tests were retained in the analysis rather than removing observations to obtain equal group sizes. Mechanical parameters are reported as mean ± standard deviation unless otherwise stated. Because both replicate numbers and variances differed among conditions, inferential comparisons were performed using Welch’s ANOVA followed by Games–Howell post hoc testing.

All data processing, statistical analysis, and figure generation were performed using custom Python scripts (Python 3.12.13; Python Software Foundation, Wilmington, DE, USA) with NumPy 2.5.4, Pandas 3.0.6, SciPy 1.18.1, and Matplotlib 3.11.2. Microsoft Excel (Microsoft Corporation, Redmond, WA, USA) was used for data organization and verification.

### 2.6. Stress Relaxation Analysis

Short-term stress relaxation was incorporated into the tensile loading sequence before rupture. Each intact O-ring was loaded to an intermediate deformation, held at constant crosshead position while the force decay was recorded, and then reloaded until rupture. Each relaxation curve was referenced to the force at the beginning of the hold and fitted using the Kohlrausch–Williams–Watts (KWW) model. Because the measurements covered only a short finite time window, the fitted long-time force parameter was treated as an asymptotic model parameter rather than an experimentally established equilibrium force. The relaxation analysis was therefore used only for comparative characterization of the measured short-term response.

### 2.7. Mechanical Performance Indicators

To provide a comprehensive assessment of the pressure-dependent mechanical response of hydrogen-aged silicone O-rings, several complementary mechanical performance indicators were extracted from the processed tensile data. These descriptors characterize not only the ultimate tensile behavior, but also the stiffness evolution, deformation efficiency, energy absorption capability, and failure characteristics of the elastomer during loading.

#### 2.7.1. Peak Tensile Strength

The maximum tensile force measured prior to specimen rupture was recorded as the peak tensile force ($F_{max}$). Engineering tensile strength was subsequently determined using the original cross-sectional area of the O-ring:

$$\sigma_{max}=\frac{F_{max}}{2A_{0}}$$

where $F_{max}$ is the maximum recorded force, and $A_{0\;}$ is the original cross-sectional area of one O-ring strand. The factor of 2 accounts for the two load-bearing strands acting in parallel during tensile loading. This parameter represents the ultimate load-carrying capacity of the material following hydrogen exposure.

#### 2.7.2. Early-Stage Tangent Modulus

The early-stage material-normalized resistance to deformation was characterized from the slope of the engineering stress–strain response over a fixed common deformation interval. To ensure direct consistency with the corresponding structural stiffness calculation, the tangent modulus was determined by linear least-squares fitting over 0–20% engineering elongation for every specimen. The same measured data points were used for the force–displacement and stress–strain representations. The early-stage tangent modulus was therefore calculated as:

$$E_{t}=\frac{\Delta \sigma }{\Delta \varepsilon }$$

where $E_{t}$ is the initial tangent modulus, Δσ is the change in engineering tensile stress, and Δε is the corresponding change in engineering strain. Because an identical deformation interval and reference geometry were used for all specimens, this definition enables a direct comparison of the early-stage material response across the unexposed and hydrogen-exposed conditions.

#### 2.7.3. Initial Structural Stiffness

Early-stage structural stiffness was determined from the slope of the force–displacement response using the same 0–20% engineering-elongation interval employed for the tangent-modulus calculation. Structural stiffness was obtained by linear least-squares fitting as:

$$K_{t}=\frac{\Delta F}{\Delta \delta }$$

where ΔF is the change in tensile force, and Δδ is the crosshead deformation.

Using identical fitting points for both representations ensures the expected geometric relationship between the tangent modulus and structural stiffness for the fixed O-ring geometry used in this study.

#### 2.7.4. Secant Stiffness

To compare structural resistance at equivalent deformation states, secant stiffness was evaluated at fixed absolute engineering elongations rather than at fractions of each specimen’s individual failure displacement. Common engineering elongations of 20% and 40% were selected because every specimen in the dataset reached both deformation states before peak force.

For each specimen, the deformation corresponding to the selected engineering elongation was calculated from the reference O-ring geometry. The tensile force at the corresponding deformation was obtained by linear interpolation between experimentally measured points within the pre-peak loading record. Secant structural stiffness was then calculated as:

$$\mathrm{Secant}\;\mathrm{stiffness}=\frac{\mathrm{Force}\;\mathrm{at}\;a\;\mathrm{chosen}\;\mathrm{elongation}}{\mathrm{Displacement}\;\mathrm{at}\;\mathrm{that}\;\mathrm{elongation}}$$

So for 20% elongation:

$$K_{20}=\frac{F_{20}}{\delta_{20}}$$

and for 40% elongation:

$$K_{40}=\frac{F_{40}}{\delta_{40}}$$

where:

- $F_{20}\;$= tensile force at 20% elongation;
- $\delta_{20\;}\;$= crosshead displacement at 20% elongation;
- $F_{40}\;$= tensile force at 40% elongation;
- $\delta_{40}\;$= crosshead displacement at 40% elongation.

Because the same 20% and 40% elongation levels were used for every specimen, the stiffness values were compared at identical physical deformation states rather than at specimen-specific fractions of failure deformation. Tensile force at each target elongation was obtained by interpolation between experimentally measured points within the pre-peak loading record, and no extrapolation beyond the measured range was used.

#### 2.7.5. Tensile Toughness

Tensile toughness was calculated as the area under the engineering stress–strain curve up to the peak tensile response:

$$UT=\int_{0}^{\varepsilon_{f}}\sigma (\varepsilon )d\varepsilon$$

Because engineering strain is dimensionless, tensile toughness has units of stress and represents the mechanical work per unit reference material volume accumulated over the measured tensile loading path up to the selected endpoint. In the present study, it is used as an integrated measure of the combined load-bearing and deformation response prior to failure. It should not be interpreted as a direct measurement of dissipated energy because the monotonic tensile test does not separate recoverable and irreversible contributions.

#### 2.7.6. Structural Loading Work

Structural loading work was calculated directly from the force–displacement response as:

$$E=\int_{0}^{\delta_{f}}F\left(\delta \right)\;d\delta$$

where $F\left(\delta \right)\;$is the tensile force, and $\delta_{f\;}$ is the displacement corresponding to the terminal point of the processed tensile response.

The resulting quantity was reported in lbf·in and represents the external mechanical work calculated from the measured force–displacement loading path of the complete O-ring specimen. This quantity does not distinguish recoverable elastic energy from irreversible dissipation and is therefore referred to here as structural loading work rather than dissipated or absorbed energy.

For specimens of identical reference geometry and identical integration endpoints, structural loading work and tensile toughness are mathematically related representations of the same underlying loading work. They are therefore interpreted together and are not treated as independent confirmation of separate physical effects.

#### 2.7.7. Interpretation of Mechanical Descriptors

The retained mechanical descriptors were selected to characterize complementary aspects of the tensile response while minimizing redundant derived quantities. Tensile strength and ultimate elongation describe terminal load-bearing and deformation capacity, while the early-stage tangent modulus and structural stiffness characterize resistance to deformation in the initial loading regime. K20 and K40 provide structural stiffness measures at common pre-failure deformation states.

Tensile toughness and structural loading work provide integrated descriptions of the measured loading path in material-normalized and structural forms, respectively. Because these quantities are mathematically related for specimens of identical geometry and common integration endpoints, they are interpreted together rather than as independent measures of separate physical mechanisms.

### 2.8. Statistical Reliability Analysis

The variability of the mechanical response was evaluated using descriptive statistics and the coefficient of variation (CV). Differences among hydrogen exposure conditions were additionally evaluated for tensile strength, ultimate elongation, tensile toughness, and structural loading work. Welch’s one-way analysis of variance (Welch ANOVA) was applied separately to each mechanical parameter to provide a group comparison that does not require the assumption of equal variances and is suitable for the unequal sample sizes among exposure conditions. Games–Howell post hoc comparisons were subsequently used to identify pairwise differences while accounting for unequal variances and sample sizes. Statistical significance was evaluated at p<0.05. Inferential results were interpreted together with the group means, standard deviations, and overall pressure-dependent response patterns.

For each hydrogen exposure condition, the arithmetic mean and sample standard deviation were calculated for the measured mechanical descriptors as described in Section 2.5. The coefficient of variation was subsequently determined as:

$$CV=\frac{s}{\bar{x}}\times 100\%$$

where s is the sample standard deviation and is $\bar{x\;}$ the corresponding arithmetic mean. The CV provides a normalized measure of specimen-to-specimen variability, enabling comparison among mechanical parameters with different magnitudes and physical units. Lower CV values indicate greater consistency among replicate specimens, whereas higher values indicate greater dispersion in the measured mechanical response. This analysis was used to evaluate how the reproducibility of selected mechanical descriptors evolved with hydrogen exposure pressure.

### 2.9. Multivariate Analysis

To evaluate specimen-level multivariate mechanical response while minimizing mathematical redundancy among derived quantities, principal component analysis (PCA) [22] was performed using a reduced set of five primary mechanical descriptors: tensile strength, ultimate elongation, early-stage tangent modulus, secant structural stiffness at 20% elongation (K20), and secant structural stiffness at 40% elongation (K40). These quantities were selected to represent failure strength, deformation capacity, early-stage material stiffness, and structural resistance at two common pre-failure deformation states.

Descriptors that were exact geometric transformations or closely related representations of the same underlying mechanical response were not simultaneously included in the PCA. In particular, early-stage structural stiffness was excluded because it is geometrically proportional to the early-stage tangent modulus for the fixed O-ring geometry, while structural loading work was excluded from the multivariate feature set because of its direct relationship with tensile loading work and tensile toughness for specimens of identical reference geometry. This reduced feature set was used to prevent mathematically related descriptors from disproportionately weighting the multivariate analysis.

Prior to PCA, each selected descriptor was standardized to zero mean and unit variance. PCA was then applied to the standardized specimen-level dataset, and the first two principal components were retained for visualization and interpretation. The PCA score plot was used to assess whether the unexposed and hydrogen-exposed specimens occupied distinct or overlapping regions of multivariate mechanical-response space.

For exploratory visualization, K-means clustering [23] was applied to the same standardized five-descriptor dataset. The number of clusters was selected from the evaluated candidate solutions using the silhouette score. Clustering was used only as a descriptive partition of specimen-level response patterns and was not interpreted as evidence of discrete pressure-defined material states. Pearson correlation coefficients were also calculated using the same five primary mechanical descriptors. To maintain consistency between the multivariate representations, the PCA loading vectors were obtained from the same PCA used for the specimen-level score analysis rather than from a second independent PCA. The loading plot therefore identifies the relative contribution and direction of each mechanical descriptor within the PC1–PC2 space, while the correlation matrix summarizes pairwise linear relationships among the selected variables. 

### 2.10. Functional Data Analysis and Functional Principal Component Analysis

To complement the scalar mechanical descriptors, functional data analysis (FDA) was applied to the measured pre-failure force–deformation histories. Particular care was taken to ensure that specimens with different failure deformations were compared only over a domain containing experimentally measured data from every specimen. Each tensile record was restricted to the pre-peak loading branch and baseline-corrected by subtracting the median force of the first three measured loading points.

A common domain corresponding to 0–40% engineering elongation was selected for functional comparison. For the investigated O-ring geometry, this interval corresponds to 0–15.704 mm of deformation. The specimen with the lowest peak elongation reached 42.68%; consequently, all 25 specimens extended beyond the entire 0–40% analysis interval. Each baseline-corrected curve was interpolated onto a common deformation grid only within this experimentally measured domain. No post-failure values were included, and no endpoint padding or extrapolation beyond an individual specimen’s measured response was performed. Consequently, the number of contributing specimens remained constant over the complete functional-analysis interval.

Functional principal component analysis (fPCA) was subsequently applied directly to the aligned baseline-corrected force curves over the shared 0–40% elongation domain. Mean centering was applied as part of the PCA decomposition, but the individual curves were not normalized by peak force or peak deformation, and no pointwise variance scaling was applied. Thus, differences in force magnitude within the common measured deformation range were retained. The first two functional principal components were retained for visualization and interpretation. Pressure-wise mean curves and pointwise 95% confidence intervals were calculated over the same common domain using all specimens at each condition.

### 2.11. SEM Imaging and Image Acquisition

Surface morphology was examined using a Thermo Fisher Scientific Quattro S field-emission scanning electron microscope (FE-SEM, Thermo Fisher Scientific, Eindhoven, Netherlands) in low-vacuum mode without a conductive coating. SEM characterization was performed on O-rings reserved specifically for microscopy and not on specimens previously subjected to tensile testing, thereby avoiding rupture- or deformation-induced surface damage. One independent O-ring was examined at each selected SEM exposure condition, and multiple surface fields were acquired from each specimen. These image fields were treated as repeated observations from the same specimen rather than as independent specimen replicates.

SEM micrographs were acquired at 500× magnification under consistent imaging conditions for specimens exposed to 0, 500, 1000, 1500, 2500, 5000, and 7000 psi hydrogen. The 200 µm scale bar provided a calibration of approximately 0.262 µm pixel^−1^. After removal of the SEM information banner, each analyzed field was approximately 335 × 251 µm, corresponding to an analyzed area of approximately 0.084 mm^2^. The same field dimensions and image-processing procedure were applied across all exposure conditions.

Because the SEM specimens were separate from the mechanically tested specimens and the SEM pressure series was selected to provide morphological comparisons across a broad pressure range rather than to reproduce every mechanical-test pressure, the SEM analysis was interpreted as exploratory field-level morphological characterization and was not used to establish specimen-level mechanical–morphological correlations.

### 2.12. Quantitative SEM Image Analysis

SEM micrographs were analyzed using a custom Python workflow incorporating established principles of quantitative image processing and analysis for polymer electron microscopy [24,25], based primarily on NumPy, SciPy, scikit-image 0.26.0, OpenCV 5.0.0, Pandas, and Matplotlib. Images were spatially calibrated using the SEM image scale, cropped to exclude non-imaging regions, and corrected for gradual illumination variation before quantitative analysis. Contrast-limited adaptive histogram equalization (CLAHE) was used only for visualization and was not applied to images used for quantitative measurements.

**Table 1:** Summary of the principal material specifications and dimensions of the silicone O-rings investigated in this work.

Property	Silicone O-Ring
Material	Silicone rubber (commercial sealing grade)
Supplier	McMaster- Carr
Supplier part No.	9396K35
Commercial silicone grade	Parker S1138
Standard	ASTM D2000, SAE AS568 [25,26]
Hardness	70 Shore A
Inner diameter	0.984 in (25.0 mm)
Outer diameter	1.262 in (32.05 mm)
Cross-section	0.139 in (3.53 mm)
Dash size Traceability	AS568-214 [26] Material certificate with traceable lot number; cure date supplied

Bright and dark surface features were identified from their grayscale contrast relative to the dominant intensity of the surrounding silicone surface using the same matrix-referenced thresholding procedure for all images. Bright features were segmented above the matrix reference by 2.5 times the estimated matrix intensity width, while dark features were segmented below the reference by the same factor. Small isolated regions containing fewer than 12 pixels were removed during morphological cleaning, and features with an equivalent diameter below approximately 0.4 µm were excluded from object-level morphometric analysis. Segmentation sensitivity was evaluated by varying the threshold factor from 1.5 to 4.0 and by comparison with alternative automated thresholding procedures. The inferred monotonic pressure associations were sensitive to threshold choice; approximately 14.3% of the evaluated threshold variants produced a significant monotonic association for bright-area fraction, whereas none produced a significant monotonic association for dark-area fraction. The segmentation-derived quantities were therefore interpreted as exploratory field-level morphological descriptors rather than evidence of robust monotonic pressure dependence.

Individual segmented features within an SEM image were treated as morphological objects used to calculate field-level descriptors and were not considered independent experimental replicates. Accordingly, the extracted SEM descriptors were interpreted as quantitative characteristics of the analyzed surface fields and were used primarily to identify pressure-associated morphological patterns rather than to establish population-level material variability.

Texture and multiscale surface organization were characterized using gray-level co-occurrence matrix (GLCM) descriptors, power spectral density (PSD), and box-counting fractal dimension. Spatial organization of the segmented bright and dark features was evaluated using Ripley’s L-function [26]. Each connected segmented feature was represented by its centroid, and the full cropped quantitative SEM field (1280 × 960 pixels; approximately 335.08 × 251.31 µm, corresponding to approximately 0.0842 mm^2^) was used as the rectangular observation window. A translation/Ohser edge correction was applied to account for boundary effects.

A separate complete-spatial-randomness (CSR) reference was generated for every pressure condition and feature class. For each observed spatial pattern, 199 Monte Carlo CSR realizations were generated by uniformly distributing the same number of points as the observed pattern within the same rectangular observation window. Ripley’s L-function was evaluated over 40 radial distances extending from approximately 1.57 to 62.83 µm. The 2.5th and 97.5th percentiles of the simulated L-functions at each distance were used to define the corresponding pointwise 95% CSR bounds. Thus, each observed feature pattern was evaluated relative to a CSR reference having the same point count and observation geometry rather than against a single common envelope.

Global image topology was additionally characterized using Minkowski functionals over 32 grayscale thresholds. The Euler characteristic was interpreted as the number of connected foreground components minus the number of enclosed holes in the thresholded image. A positive Euler characteristic therefore indicates that connected components outnumber holes, whereas negative values indicate that holes outnumber connected components. This quantity was used strictly as a descriptor of threshold-dependent image topology and was not interpreted as evidence of physical connectivity of the polymer network.

## 3. Results and Discussion

### 3.1. Pressure-Dependent Tensile Response

**Figure 3:**
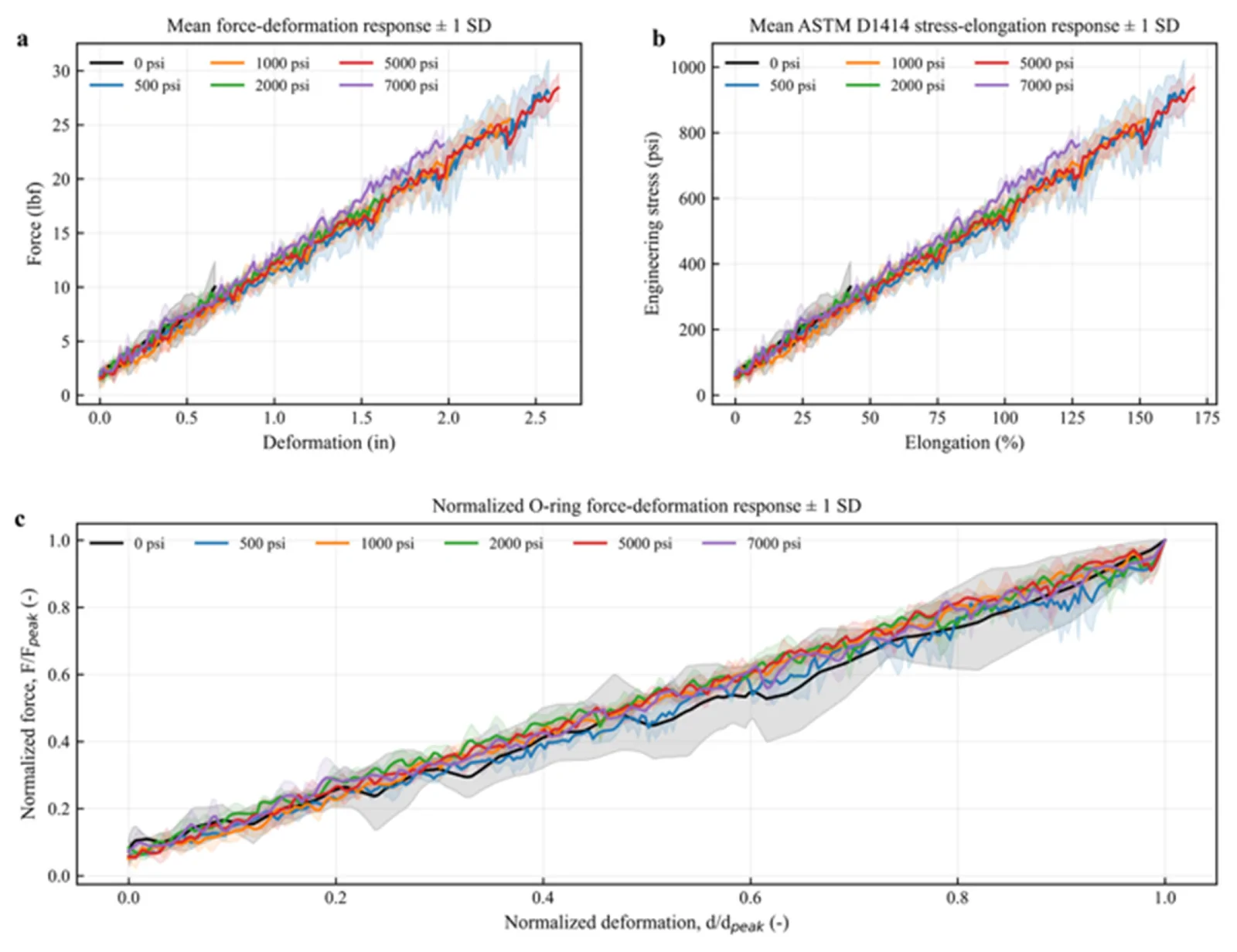
Global tensile response of commercial silicone O-rings for the unexposed control (0 psi) and following hydrogen exposure at 500–7000 psi. (**a**) Mean force–deformation curves with ±1 standard deviation envelopes. (**b**) Mean engineering stress–elongation responses. (**c**) Normalized force–deformation curves obtained by scaling force and deformation to their respective specimen-level peak values. The normalized representation compares loading-path shape independently of absolute response magnitude and failure deformation. The shaded regions represent ±1 standard deviation around the corresponding mean curves, indicating specimen-to-specimen variability.

Figure 3 presents the mean force–deformation, engineering stress–elongation, and normalized force–deformation responses of the unexposed silicone O-rings and specimens following hydrogen exposure at pressures from 500 to 7000 psi. The inclusion of the 0 psi condition provides a direct unexposed reference for evaluating exposure-associated changes in the tensile response. These representations describe the structural response of the complete O-rings, the corresponding engineering stress–strain response, and the deformation pathway after normalization of the absolute response magnitude.

Figure 3a shows that all conditions exhibited a continuous increase in tensile force until rupture, without abrupt force loss during the principal loading stage. Compared with the unexposed 0 psi group, the hydrogen-exposed specimens generally sustained deformation over a larger displacement range before reaching peak force, with the greatest extension observed for several of the 500, 1000, and 5000 psi specimens. The exposed groups did not follow a monotonic progression with pressure; rather, the force–deformation trajectories varied non-monotonically across the investigated conditions. The 7000 psi group exhibited lower failure deformation than several lower-pressure exposed groups while still showing a different response from the unexposed reference. The standard-deviation envelopes further indicate appreciable specimen-to-specimen variability, particularly for the 0 psi control and near the terminal region of loading. These observations therefore support an exposure-associated change in deformability but do not indicate a simple monotonic deterioration or improvement with increasing hydrogen pressure.

The engineering stress–elongation curves in Figure 3b show trends consistent with the force–deformation response. The unexposed specimens reached failure at lower mean elongation than several hydrogen-exposed groups, whereas the exposed specimens generally extended over a larger strain range before rupture. Differences among the exposed groups became more apparent at larger elongations, with the 7000 psi condition showing lower deformation capacity than the 500, 1000, and 5000 psi groups. Accordingly, the addition of the unexposed control indicates that the principal exposure-associated change was not a uniform reduction in mechanical response but a non-monotonic change in deformation and failure behavior across the investigated pressure conditions.

Figure 3c compares the response shapes after normalizing force and deformation to the respective specimen-level peak values. Considerable overlap is observed among the normalized curves, including the unexposed condition, over much of the normalized loading history. This indicates that the general shape of the tensile loading pathway was less sensitive to exposure condition than the absolute failure force and deformation. Importantly, normalization removes differences in response magnitude and failure deformation; therefore, the observed overlap is interpreted only as similarity in normalized curve shape and not as evidence of mechanical equivalence among the conditions.

### 3.2. Ultimate Tensile Performance Following Hydrogen Exposure

**Figure 4:**
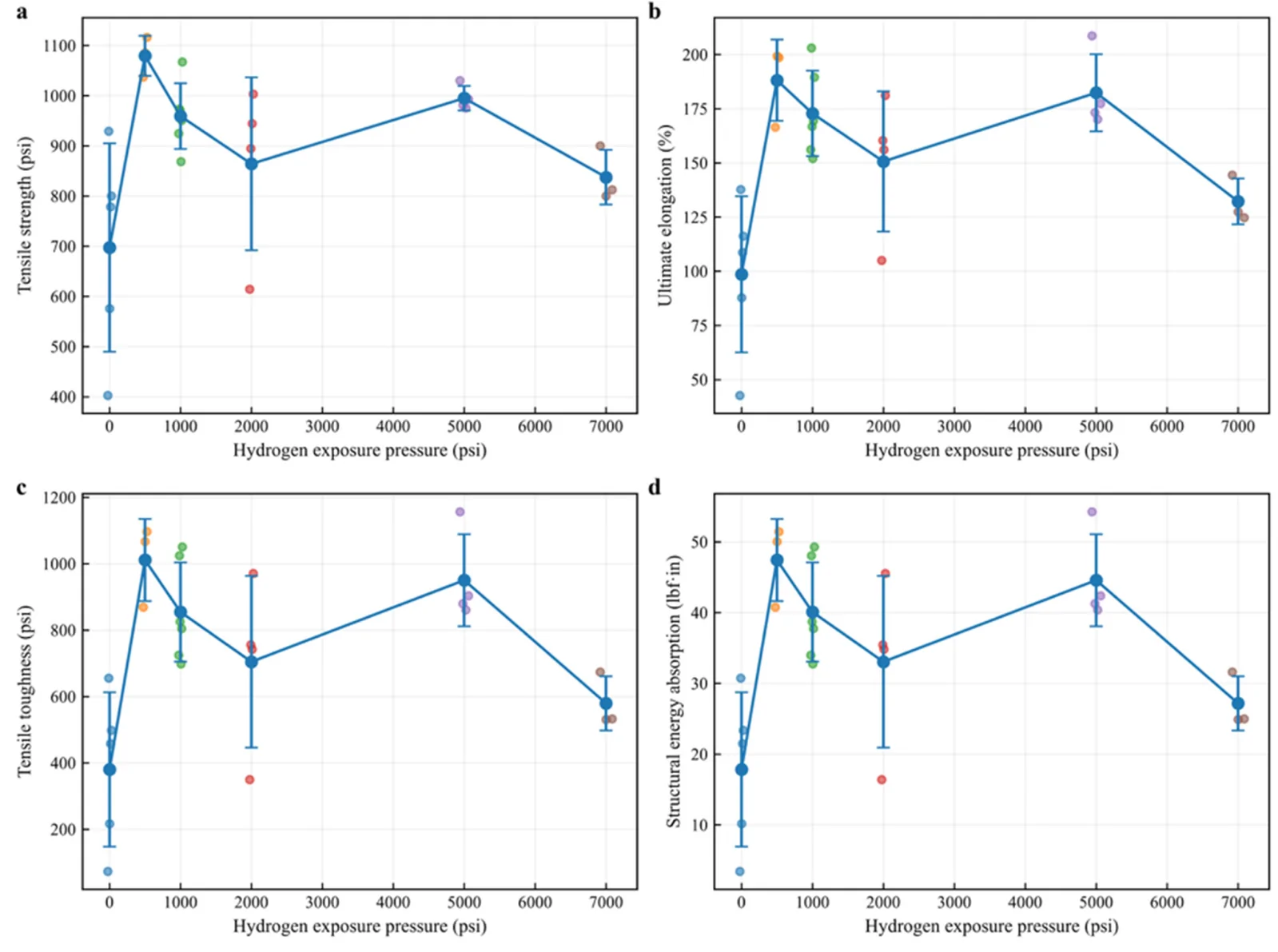
Mechanical response of commercial silicone O-rings for the unexposed control and following hydrogen exposure. (**a**) Tensile strength. (**b**) Ultimate elongation at break. (**c**) Tensile toughness calculated by integration of the engineering stress–strain response. (**d**) Structural loading work calculated by integration of the force–displacement response. Symbols represent group means and error bars indicate ±1 standard deviation. Sample sizes were n = 5, 3, 6, 4, 4, and 3 for the 0, 500, 1000, 2000, 5000, and 7000 psi conditions, respectively. Overall group differences were evaluated using Welch ANOVA followed by Games–Howell post hoc comparisons. The colored dots represent individual specimen measurements at each hydrogen exposure pressure, while the blue markers and connecting lines indicate group means, with error bars representing ±1 standard deviation.

Having established the response of the unexposed material and the hydrogen-exposed conditions in Figure 3, the principal failure-related mechanical properties were compared directly. Figure 4 summarizes the tensile strength, ultimate elongation, tensile toughness, and structural loading work for the 0 psi control and the five hydrogen exposure pressures. The inclusion of the unexposed condition enables the direct quantification of exposure-associated differences relative to the initial material state.

Figure 4a,b shows that the inclusion of the unexposed control substantially changes the interpretation of the tensile response. Mean tensile strength was 697 ± 208 psi (4.81 ± 1.43 MPa) for the 0 psi group and increased to 1079 ± 40 psi (7.44 ± 0.27 MPa) at 500 psi. The mean values were 959 psi at 1000 psi, 864 psi at 2000 psi, 995 psi at 5000 psi, and 837 psi at 7000 psi. Welch ANOVA identified a significant overall group effect for tensile strength (*p* = 0.0073). Games–Howell analysis identified a significant difference between the 500 and 7000 psi groups (*p* = 0.024), whereas the pairwise comparisons between the unexposed control and individual exposed groups were not statistically significant. Thus, the strength data indicate substantial group-level variation but do not support a monotonic pressure-dependent increase or decrease relative to the unexposed material.

Ultimate elongation exhibited a clearer difference relative to the unexposed reference. Mean elongation increased from 98.6 ± 36.0% at 0 psi to 188.1% at 500 psi, 172.8% at 1000 psi, 150.6% at 2000 psi, and 182.3% at 5000 psi before decreasing to 132.2% at 7000 psi. Welch ANOVA indicated a significant overall group effect (*p* = 0.0068). The 0 psi group showed significantly lower elongation than the 500 psi (*p* = 0.026), 1000 psi (*p* = 0.044), and 5000 psi (*p* = 0.027) groups. In addition, elongation at 7000 psi was significantly lower than at 1000 psi (*p* = 0.040) and 5000 psi (*p* = 0.038). These results show that several hydrogen-exposed groups exhibited greater deformation capacity than the unexposed control, followed by a reduction at the highest investigated pressure relative to selected lower-pressure exposed conditions.

The energy-related properties shown in Figure 4c,d followed a similar non-monotonic pattern. Mean tensile toughness increased from 380 ± 233 psi (2.62 ± 1.61 MPa) for the unexposed group to 1011 psi at 500 psi, 854 psi at 1000 psi, 704 psi at 2000 psi, and 950 psi at 5000 psi before decreasing to 579 psi at 7000 psi. Structural loading work correspondingly increased from 17.8 ± 10.9 lbf·in at 0 psi to 47.4 lbf·in at 500 psi, 40.1 lbf·in at 1000 psi, 33.0 lbf·in at 2000 psi, and 44.6 lbf·in at 5000 psi, followed by a decrease to 27.2 lbf·in at 7000 psi. Welch ANOVA showed significant overall group effects for both quantities (*p* = 0.0065). Games–Howell comparisons showed significantly lower values for the 0 psi group than the 500, 1000, and 5000 psi groups, while the 5000 psi group also exceeded the 7000 psi group. Because tensile toughness and structural loading work are mathematically related representations of the same loading work when specimen geometry is fixed, their similar trends are interpreted together rather than as independent confirmation of two separate physical effects.

The overall response is therefore more appropriately described as non-monotonic than as a progressive pressure-dependent degradation trend. Relative to the unexposed control, several exposed groups exhibited increased elongation and mechanical work to failure, while the 7000 psi condition showed lower values than selected lower-pressure exposed groups. The local increase observed at 5000 psi should not be interpreted as a characteristic pressure threshold or recurring transition because only discrete pressure levels were investigated. Establishing a continuous pressure–response relationship would require additional measurements at more closely spaced pressure intervals.

The observed increase in deformation capacity and loading work after exposure at several pressures indicates greater post-exposure deformability relative to the unexposed control. Such behavior may be consistent with reversible changes in polymer-chain mobility, gas sorption, or other hydrogen–polymer interactions reported for elastomeric materials; however, the present tensile measurements do not directly establish a molecular mechanism. Likewise, the reduction in elongation and energy-related properties at 7000 psi relative to several lower-pressure groups may reflect competing exposure- and decompression-related processes, but these processes were not independently quantified. Accordingly, the present results are interpreted as experimentally observed changes in macroscopic mechanical response, while mechanistic explanations remain hypotheses requiring complementary measurements such as spectroscopy, dynamic mechanical analysis, sorption, swelling, or controlled desorption characterization.

### 3.3. Evolution of Initial and Secant Stiffness

**Figure 5:**
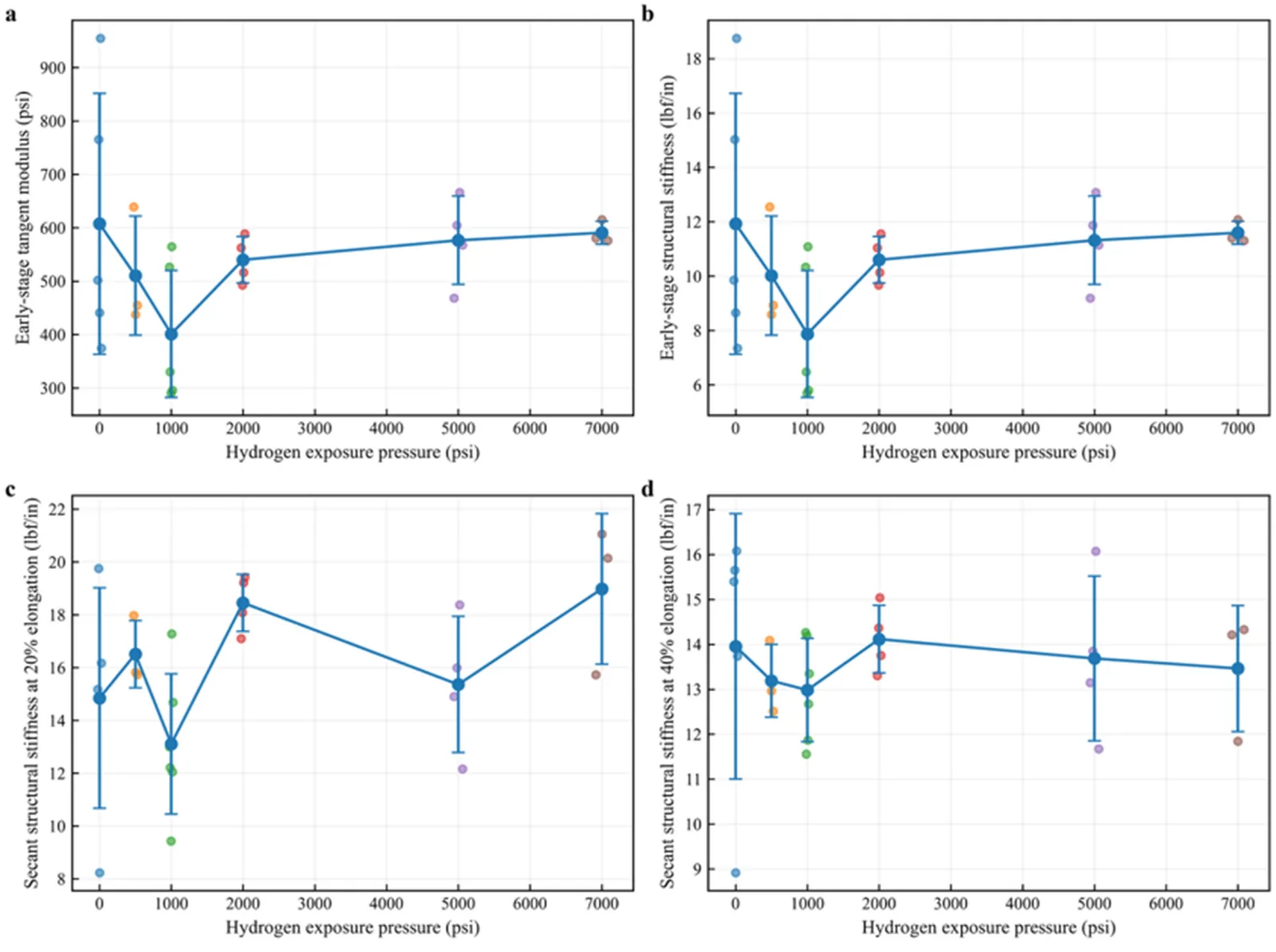
Stiffness-related mechanical response of commercial silicone O-rings for the unexposed control and following hydrogen exposure. (**a**) Early-stage tangent modulus calculated over the common 0–20% engineering-elongation interval. (**b**) Early-stage structural stiffness calculated from the corresponding force–displacement data over the same 0–20% interval. (**c**) Secant structural stiffness at 20% of engineering elongation. (**d**) Secant structural stiffness at 40% of engineering elongation. Individual markers represent specimens, while the larger symbols and error bars represent group means ±1 standard deviation. The different colored dots represent individual specimen measurements at each hydrogen exposure pressure, while the larger blue markers and connecting lines indicate group means, with error bars representing ±1 standard deviation.

While Figure 4 describes the failure-related mechanical response, Figure 5 evaluates resistance to deformation at common pre-failure states. The early-stage tangent modulus and structural stiffness were recalculated over the identical 0–20% engineering-elongation interval, while secant structural stiffness was evaluated at 20% ($K_{20}$) and 40% ($K_{40}$).

This approach ensures that specimens are compared at equivalent physical deformation states and that the tangent-modulus and structural-stiffness calculations are geometrically consistent.

Figure 5a,b shows that the recalculated early-stage tangent modulus and structural stiffness exhibit identical pressure-dependent trends, consistent with their geometric relationship. Mean early-stage tangent modulus was approximately 608 psi for the unexposed condition, decreased to 510 psi at 500 psi and 401 psi at 1000 psi, and subsequently increased to approximately 540, 577, and 591 psi at 2000, 5000, and 7000 psi, respectively. The corresponding mean structural stiffness changed proportionally from 11.93 lbf/in at 0 psi to 10.02 and 7.87 lbf/in at 500 and 1000 psi, followed by 10.60, 11.32, and 11.60 lbf/in at 2000, 5000, and 7000 psi, respectively. Thus, the early-stage response exhibited a non-monotonic reduction at the lower exposure conditions followed by recovery toward the unexposed mean at higher pressures. The proportional trends confirm that the previously observed difference between tangent modulus and structural stiffness resulted from the analysis procedure rather than from distinct physical evolution of these quantities.

The common-state secant-stiffness results provide a complementary description of the nonlinear loading response. At 20% elongation, mean K20 was 14.85 lbf/in for the unexposed control and 16.51, 13.11, 18.46, 15.36, and 18.98 lbf/in for the 500, 1000, 2000, 5000, and 7000 psi groups, respectively. In contrast, K40 varied over a substantially narrower range: the unexposed mean was 13.95 lbf/in, while the hydrogen-exposed means ranged from approximately 12.98 to 14.11 lbf/in. No monotonic relationship with hydrogen pressure was observed. The relatively narrow range of K40 indicates that resistance to deformation at this common intermediate elongation was comparatively similar among the investigated conditions, even though substantially larger differences were observed in terminal elongation and loading work to failure.

Overall, the corrected stiffness analysis demonstrates that the magnitude of the exposure-associated response depends on the deformation regime considered. Early-stage stiffness showed a non-monotonic decrease followed by recovery toward the unexposed value, whereas structural stiffness at the common 40% elongation state remained within a comparatively narrow range. In contrast, the failure-related quantities in Figure 4, particularly ultimate elongation, tensile toughness, and structural loading work, exhibited substantially larger differences relative to the unexposed condition. This distinction is mechanically plausible because K40 describes resistance at a specified pre-failure deformation state, whereas ultimate elongation and integrated loading work additionally depend on how far the specimen continues to deform before rupture. The present tensile measurements establish this difference in macroscopic response but do not identify the molecular mechanism responsible for it.

### 3.4. Integrated Mechanical Evolution Across Hydrogen Exposure Pressures

**Figure 6:**
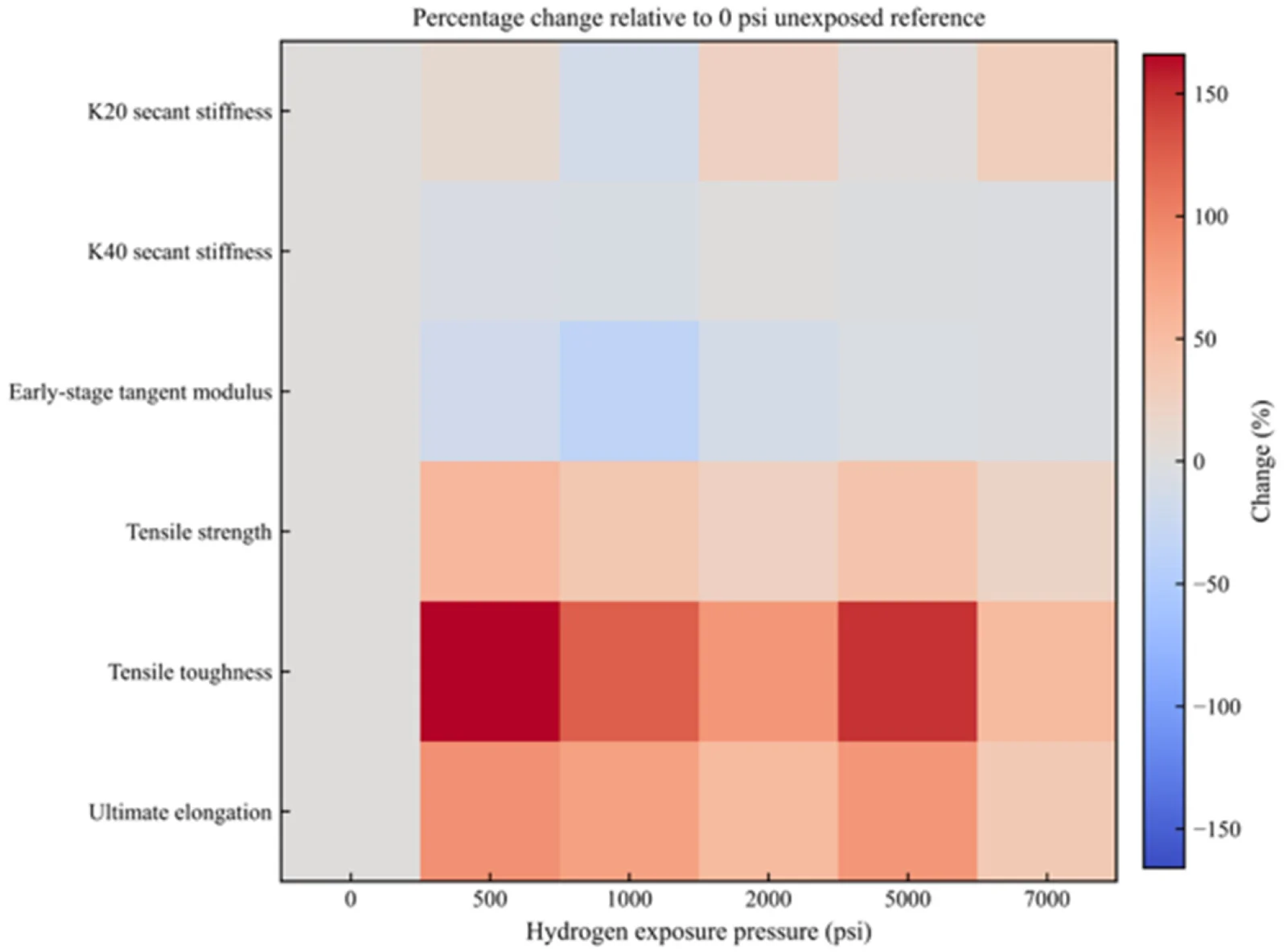
Integrated visualization of changes in selected non-redundant mechanical descriptors relative to the unexposed 0 psi reference. The heatmap shows percentage differences in K20 secant stiffness, K40 secant stiffness, early-stage tangent modulus, tensile strength, tensile toughness, and ultimate elongation relative to the corresponding unexposed group mean. Positive values indicate values above the unexposed reference and negative values indicate values below it. The heatmap is descriptive and does not independently represent statistical significance.

Following the individual evaluation of failure-related properties in Figure 4, the corrected common-state stiffness descriptors in Figure 5 and Figure 6 summarize the relative changes in six selected mechanical quantities using the unexposed 0 psi condition as the reference. To avoid over-representation of mathematically related variables, the heatmap includes K20, K40, early-stage tangent modulus, tensile strength, tensile toughness, and ultimate elongation. The 0 psi values are therefore defined as zero percentage change, while positive and negative values indicate higher and lower group means, respectively, relative to the unexposed reference.

The heatmap demonstrates that the different mechanical response categories did not change uniformly following hydrogen exposure. Relative to the unexposed condition, tensile strength increased by approximately 54.8% at 500 psi, 37.6% at 1000 psi, 24.0% at 2000 psi, 42.7% at 5000 psi, and 20.1% at 7000 psi. Ultimate elongation exhibited larger relative increases of approximately 90.8%, 75.2%, 52.8%, 84.9%, and 34.1%, respectively. Tensile toughness showed the largest relative differences, increasing by approximately 166.0% at 500 psi, 124.8% at 1000 psi, 85.3% at 2000 psi, 150.0% at 5000 psi, and 52.4% at 7000 psi.

The stiffness-related quantities exhibited substantially smaller and more variable changes. Early-stage tangent modulus decreased relative to the unexposed mean at all exposed conditions, with the largest reduction occurring at 1000 psi (approximately −34.0%), followed by progressively smaller differences at 2000, 5000, and 7000 psi. K20 varied non-monotonically from approximately −11.7% to +27.9% relative to the unexposed reference. In contrast, K40 remained particularly stable, with group-mean differences ranging only from approximately −7.0% to +1.2% across all exposed conditions.

Overall, Figure 6 highlights a clear distinction between changes in failure-related capacity and resistance to deformation at common pre-failure states. Ultimate elongation and tensile toughness exhibited comparatively large exposure-associated differences, whereas K40 remained close to the unexposed reference throughout the investigated pressure range. The response was nevertheless non-monotonic, and the heatmap should be interpreted together with the group variability and inferential statistical results presented in Figure 4 and Figure 5 rather than as evidence of a continuous pressure–response relationship.

### 3.5. Statistical Variability and Multivariate Analysis

**Figure 7:**
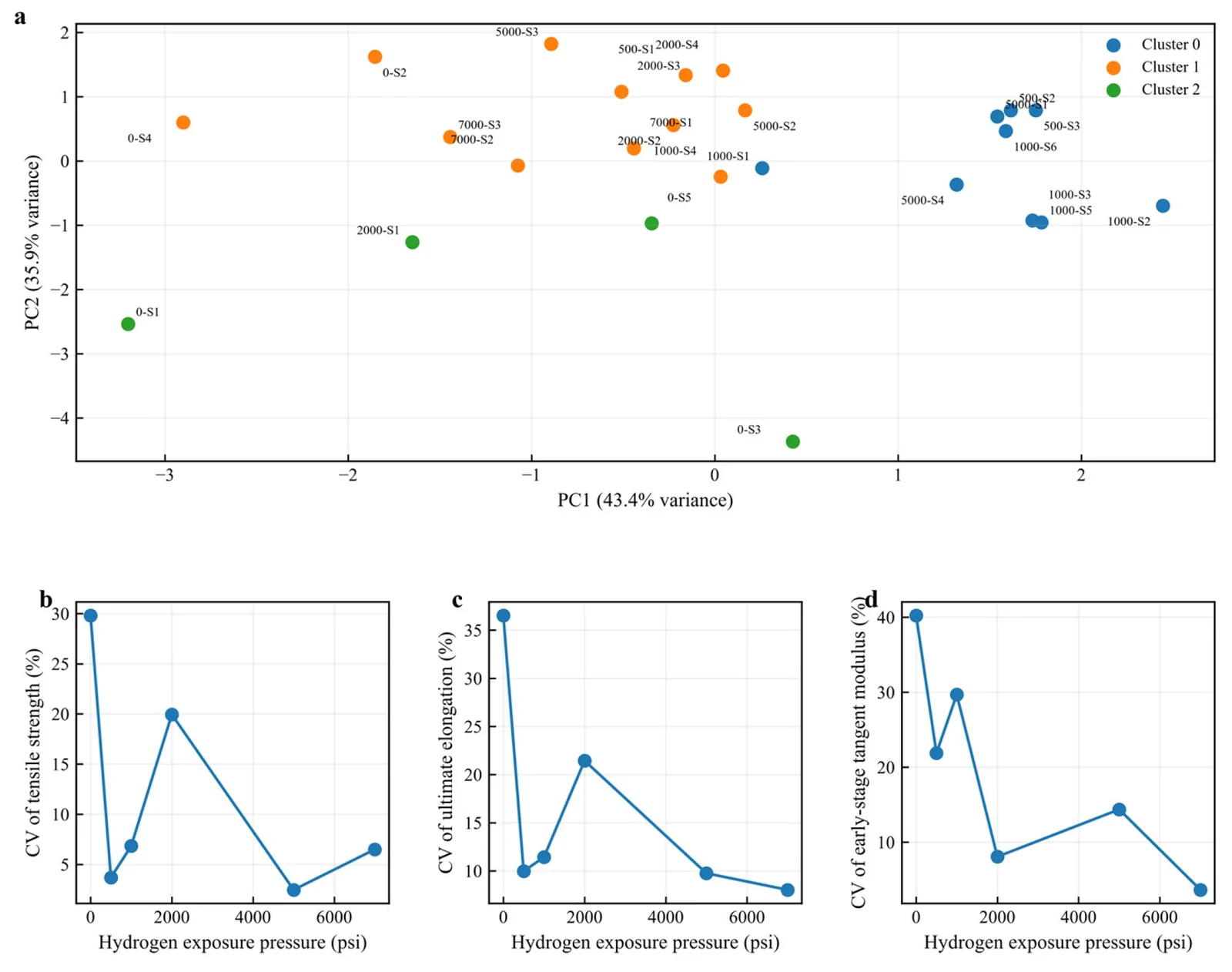
Statistical variability and multivariate characterization of the tensile response for the unexposed control and hydrogen-exposed groups. (**a**) PCA score plot based on the standardized five-descriptor mechanical feature set comprising tensile strength, ultimate elongation, early-stage tangent modulus, K20, and K40; colors indicate the exploratory K-means partition and specimen labels identify exposure condition. (**b**) Coefficient of variation (CV) of tensile strength. (**c**) CV of ultimate elongation. (**d**) CV of early-stage tangent modulus.

Following the evaluation of the pressure-dependent group means in Figure 4, Figure 5, Figure 6 and Figure 7 examines specimen-to-specimen variability and multivariate organization using the reduced five-descriptor mechanical feature set. The PCA incorporates tensile strength, ultimate elongation, early-stage tangent modulus, K20, and K40, thereby representing complementary aspects of failure capacity, deformability, and pre-failure stiffness while excluding mathematically redundant quantities.

The first two principal components in Figure 7a account for approximately 79.3% of the total variance, with PC1 explaining 43.4% and PC2 explaining 35.9%. The exploratory K-means analysis identified three clusters, with a silhouette score of approximately 0.295. The clusters contain specimens from multiple pressure conditions and therefore do not correspond to uniquely defined hydrogen exposure levels. Considerable overlap is evident among the exposed groups, while several unexposed specimens occupy more dispersed regions of the PCA space. Accordingly, the clustering is interpreted only as an exploratory description of specimen-level multivariate similarity and not as evidence of discrete pressure-specific mechanical states.

The CV analysis further shows that specimen-to-specimen variability depends strongly on both the descriptor and exposure condition. For tensile strength, the unexposed group exhibited the greatest CV at approximately 29.8%, followed by the 2000 psi group at approximately 19.9%; the remaining exposed groups showed substantially lower relative variability. Ultimate elongation similarly showed its greatest variability in the unexposed condition (36.5%), followed by 2000 psi (21.4%). Early-stage tangent modulus exhibited the largest relative dispersion of the three descriptors, with a CV of approximately 40.2% for the unexposed group and 29.7% at 1000 psi, while variability decreased to approximately 3.6% at 7000 psi. These results emphasize that differences in group means should be interpreted together with the substantial baseline variability observed for several mechanical descriptors.

**Figure 8:**
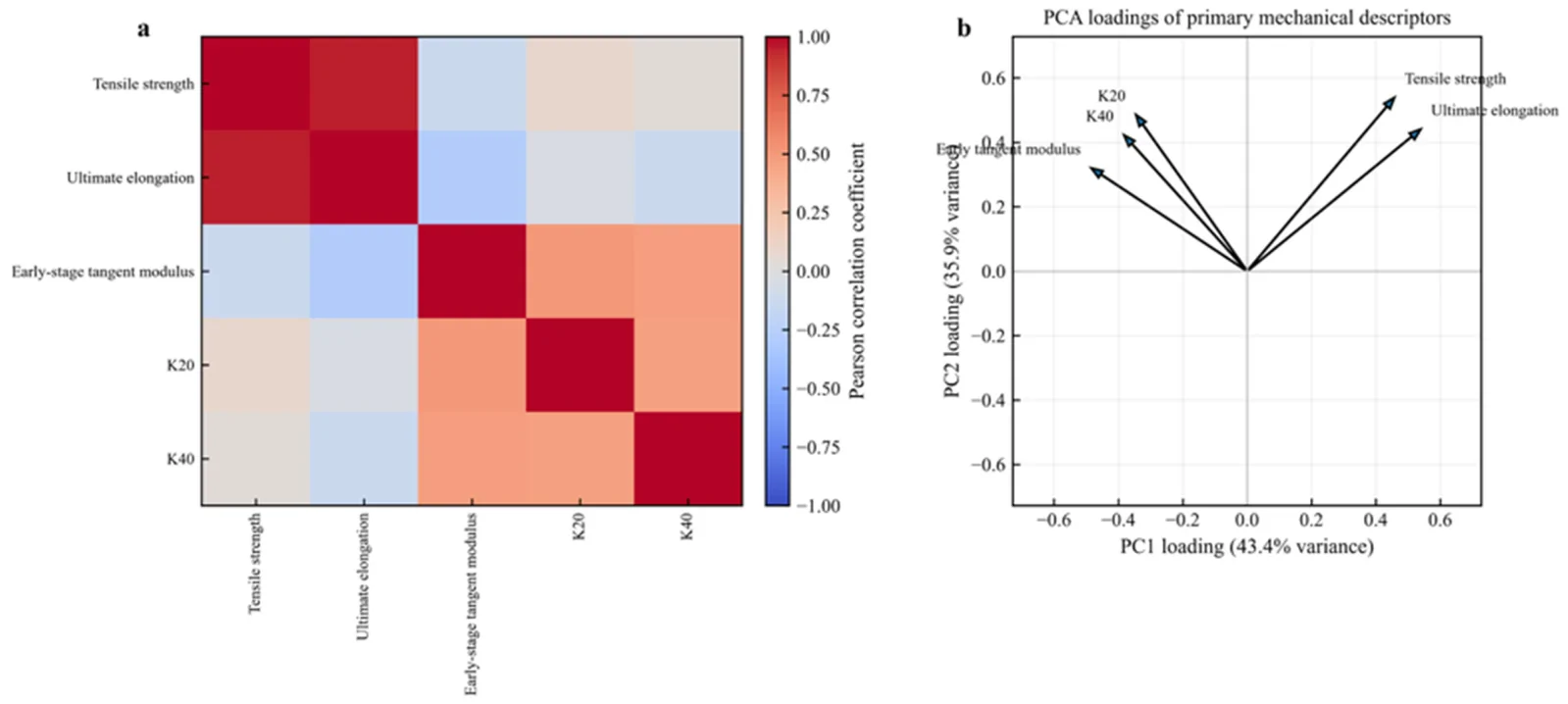
Relationships among the five primary mechanical descriptors used in the multivariate analysis. (**a**) Pearson correlation matrix for tensile strength, ultimate elongation, early-stage tangent modulus, K20, and K40. (**b**) PCA loading plot showing the contribution and direction of the same five descriptors in the PC1–PC2 space. The loading plot corresponds to the PCA score representation shown in Figure 7a and therefore uses the same explained-variance values (PC1: 43.4%; PC2: 35.9%).

Relationships among the five selected descriptors are examined in Figure 8 using Pearson correlation analysis and the loading vectors from the same PCA used in Figure 7a.

The correlation matrix in Figure 8a shows a strong positive relationship between tensile strength and ultimate elongation (r ≈ 0.95), indicating that specimens sustaining higher tensile strength in this dataset also generally reached greater deformation before failure. In contrast, early-stage tangent modulus showed weak negative relationships with tensile strength (r ≈ −0.13) and ultimate elongation (r ≈ −0.28). The stiffness-related descriptors were moderately positively associated with one another: early-stage tangent modulus correlated with K20 at approximately r = 0.51 and with K40 at approximately r = 0.48, while K20 and K40 showed a correlation of approximately r = 0.46. These relationships are interpreted descriptively and do not establish causation or a common underlying molecular mechanism.

The PCA loading plot in Figure 8b clarifies the contributions of the selected descriptors to the multivariate response. Tensile strength and ultimate elongation load positively on PC1, with loadings of approximately +0.46 and +0.54, respectively, whereas early-stage tangent modulus, K20, and K40 load negatively on PC1 at approximately −0.48, −0.35, and −0.38. PC1 therefore primarily contrasts greater failure strength and deformability against greater stiffness-related resistance within the present dataset. All five descriptors contribute positively to PC2, with tensile strength, K20, ultimate elongation, K40, and early-stage tangent modulus showing loadings of approximately +0.54, +0.49, +0.44, +0.42, and +0.32, respectively. Because the loading structure is based on the same PCA shown in Figure 7a, no second independent PCA or additional explained-variance estimate is required.

Taken together, Figure 7 and Figure 8 indicate that the specimen-level mechanical response is governed by coordinated variation in failure-related and stiffness-related quantities rather than by a single pressure-dependent variable. The unexposed group displays comparatively high variability, while the hydrogen-exposed groups remain substantially overlapping in the reduced multivariate space. These analyses provide a descriptive summary of correlated mechanical behavior and complement, but do not replace, the direct statistical comparisons of the individual mechanical properties.

### 3.6. Functional Analysis of the Common Pre-Failure Tensile Response

**Figure 9:**
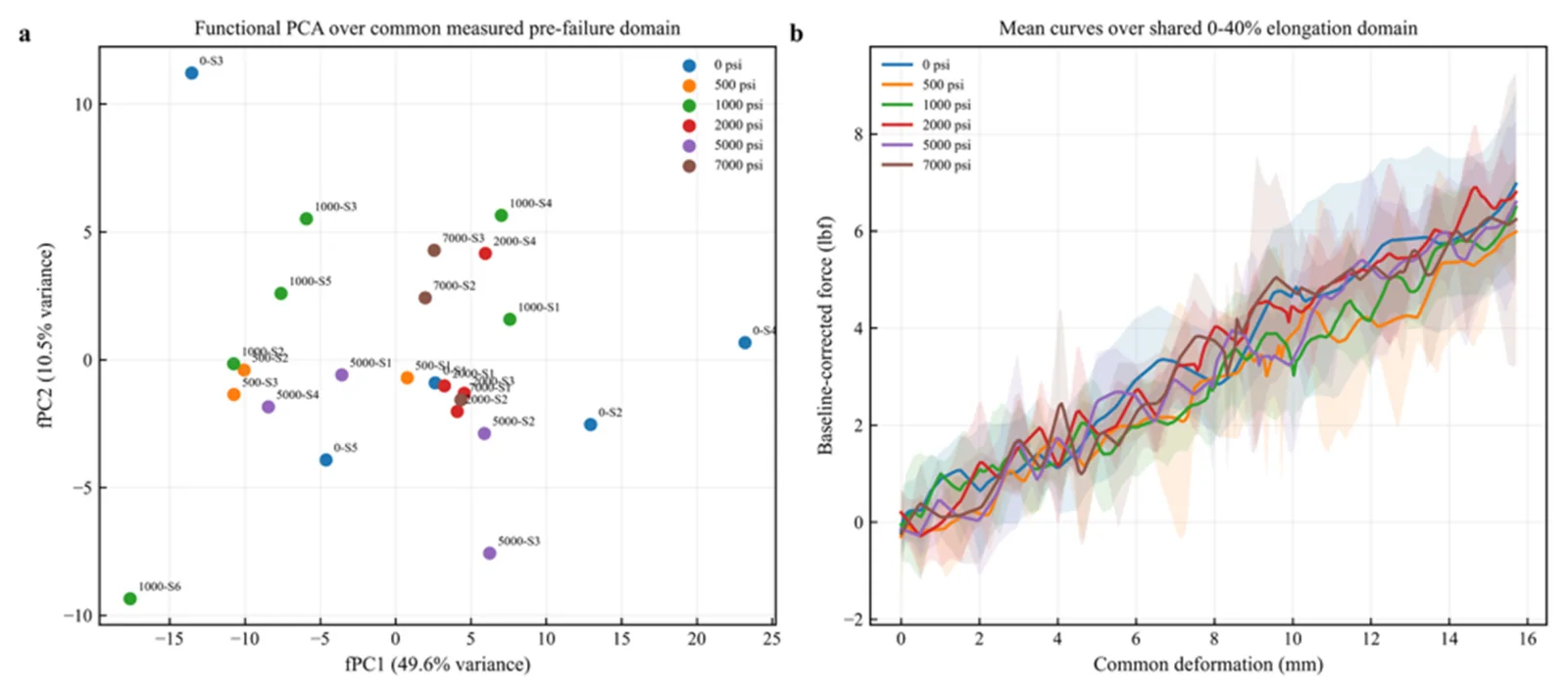
Functional characterization of the tensile response of commercial silicone O-rings over a common measured pre-failure deformation domain. (**a**) Functional principal component analysis (fPCA) of baseline-corrected force–deformation curves over 0–40% engineering elongation; fPC1 and fPC2 explain 49.6% and 10.5% of the total variance, respectively. (**b**) Pressure-wise mean baseline-corrected force–deformation curves over the shared 0–40% elongation domain. The solid colored lines represent the mean responses for each hydrogen exposure condition, while the corresponding shaded regions indicate pointwise 95% confidence intervals. All specimens contributed measured data throughout the complete interval; no post-failure padding or extrapolation was used.

While the preceding analyses quantify individual mechanical descriptors and their statistical relationships, functional analysis was performed to evaluate variation in the tensile response over a strictly shared measured pre-failure domain. All specimens were compared over 0–40% engineering elongation, corresponding to 0–15.704 mm deformation. The specimen with the lowest peak elongation reached 42.68%; therefore, every specimen contributes experimentally measured observations throughout the complete functional-analysis interval. This approach avoids changes in the number of contributing curves with deformation and eliminates post-failure padding or extrapolation.The functional principal component analysis and pressure-wise mean force–deformation responses over the common pre-failure domain are presented in Figure 9.

The corrected functional principal component analysis in Figure 9a shows that fPC1 and fPC2 account for 49.6% and 10.5% of the total functional variance, respectively, corresponding to 60.1% for the first two components combined. The specimen scores are broadly distributed and show substantial overlap among the pressure conditions rather than forming uniquely separated pressure-specific groups. The unexposed specimens exhibit substantial dispersion, and within-group variability is also evident for several hydrogen-exposed conditions. Accordingly, fPCA is interpreted as a descriptive representation of specimen-level curve variation over the common measured pre-failure domain and not as evidence of mechanical equivalence among conditions.

Figure 9b shows that the pressure-wise mean curves follow broadly similar increasing force–deformation trajectories over the common 0–40% elongation interval, while differences in mean force and confidence-interval width remain evident among the conditions. Because all 25 specimens contribute measured observations throughout the entire interval, these differences represent the experimentally sampled pre-failure response rather than changes caused by specimen dropout at increasing deformation. Overlap of the pointwise confidence intervals is therefore interpreted descriptively and is not considered evidence of statistical equivalence.

Overall, the corrected functional analysis indicates that differences in the common pre-failure force–deformation trajectory are smaller than the differences observed in terminal deformation and loading work to failure. This interpretation is consistent with the common-state stiffness analysis in Figure 5, particularly the relatively narrow range of K40, whereas Figure 4 demonstrates substantially larger differences in ultimate elongation, tensile toughness, and structural loading work. Importantly, Figure 9 characterizes only the response over the shared 0–40% elongation interval and therefore does not assess or imply equivalence in mechanical behavior approaching rupture or at failure.

### 3.7. Short-Term Viscoelastic Relaxation

Short-term stress–relaxation measurements were performed to provide a complementary assessment of the time-dependent mechanical response of the unexposed and hydrogen-exposed silicone O-rings. The unexposed 0 psi specimens were obtained from the same manufacturing batch and lot as the hydrogen-exposed specimens and were evaluated using the same stress–relaxation procedure, thereby providing a direct reference condition for comparison. Representative relaxation behavior was described using the Kohlrausch–Williams–Watts (KWW) model. Among the hydrogen-exposed conditions, only modest pressure-associated variation was observed in the short-term relaxation response, and no consistent monotonic pressure dependence was identified across the investigated exposure pressures. Inclusion of the identically tested 0 psi control enables the relaxation behavior of the exposed specimens to be evaluated relative to the unexposed material state rather than solely through comparisons among the exposed groups.

The stress–relaxation measurements are therefore interpreted as a complementary comparison of short-term time-dependent behavior between the unexposed reference and the hydrogen-exposed conditions. Because the relaxation measurements represent a limited short-duration observation window, the fitted KWW parameters are used for comparative characterization of the measured relaxation response and are not interpreted as establishing long-term equilibrium viscoelastic behavior.

### 3.8. Engineering Implications for Hydrogen Seal Reliability

From an engineering perspective, the results support a response-regime-based approach to evaluating hydrogen-exposed silicone seals. Mechanical screening should distinguish among properties describing pre-failure deformation resistance, terminal failure behavior, and the overall loading path rather than treating any single descriptor as a surrogate for overall material integrity. Such a multi-parameter assessment provides a more defensible basis for material screening and subsequent seal-level qualification.

The present tensile measurements characterize material-level mechanical response but do not directly quantify sealing contact pressure, leakage, permeability, or component service life and therefore should not be interpreted as demonstrating seal-level performance or safety. Instead, they provide a comparative basis for future seal-qualification studies incorporating compression, leakage, sorption, and cyclic-pressure testing.

The present response should also be distinguished from previous silicone O-ring studies conducted under cyclic hydrogen service conditions. Earlier work on filled silicone O-rings used 100 pressure cycles at 70 MPa and 30 °C and focused primarily on leakage and extrusion-fracture behavior, while long-term durability investigations reported silicone O-ring performance over as many as 5500 pressure cycles at 70 MPa under specific sealing configurations. In contrast, the present study uses a 192 h fixed-pressure exposure protocol over 500–7000 psi (approximately 3.4–48 MPa) followed by post-exposure mechanical characterization. Accordingly, direct numerical comparison of tensile properties with those cycling studies is not possible because the loading histories and measured outputs differ.

Accordingly, the practical contribution of the present mechanical dataset is the demonstration that different response regimes should be evaluated separately during hydrogen-service qualification. Intermediate-deformation resistance should not be used as a surrogate for the preservation of failure behavior; both should be considered together with seal-level performance measures when assessing material suitability.

Although the present work does not introduce a new silicone formulation, the observed response also provides direction for future material development. Formulation strategies may be evaluated not only for the preservation of stiffness, but also for their ability to retain failure-related strength, deformation capacity, and energy absorption while limiting localized morphological changes under hydrogen exposure. Because filler chemistry, filler loading, crosslink density, and other proprietary formulation variables were not independently controlled in the present study, specific material modifications cannot be prescribed from these results; instead, the findings provide performance targets against which future silicone formulations can be evaluated.

## 4. SEM Characterization of Surface Morphology Following Hydrogen Exposure

### 4.1. Qualitative SEM Observations

**Figure 10:**
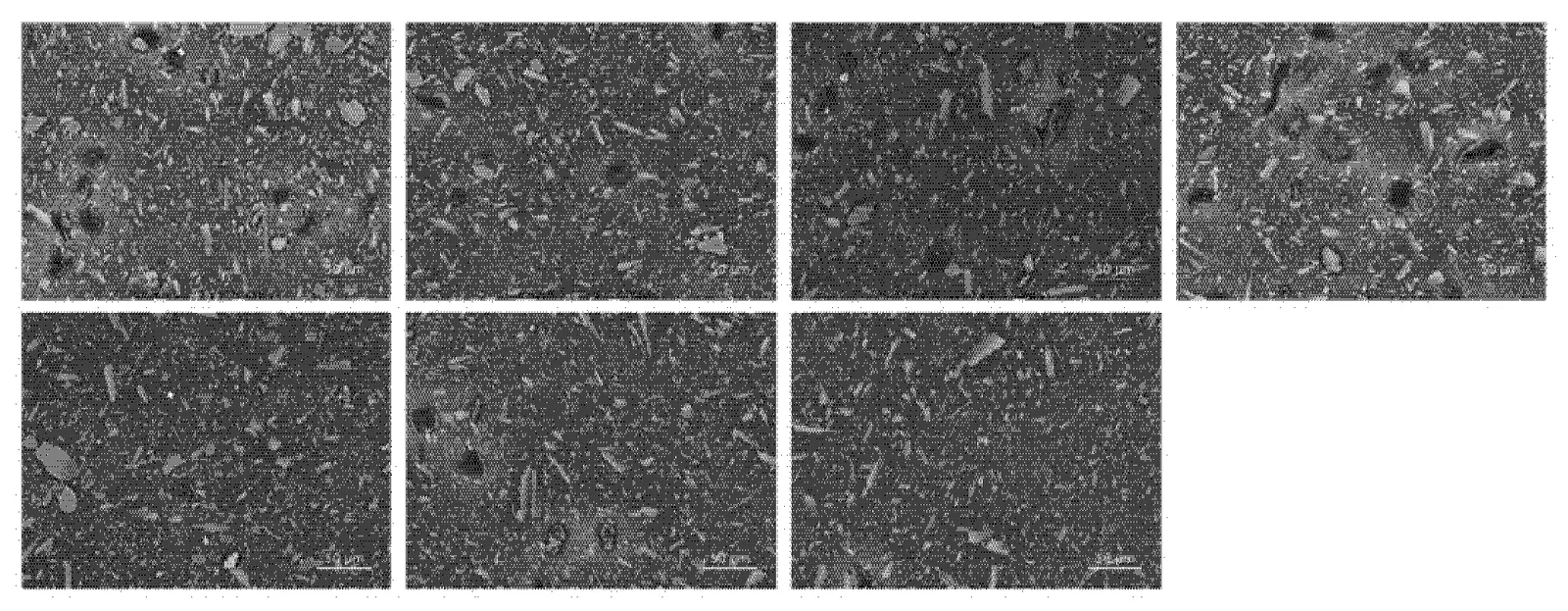
Representative scanning electron microscopy (SEM) micrographs of commercial silicone O-ring surfaces before hydrogen exposure (0 psi, unexposed) and after conditioning at 500, 1000, 1500, 2500, 5000, and 7000 psi. The images show visually continuous surfaces together with pressure-associated differences in the morphology, distribution, and spatial organization of localized bright high-intensity and dark low-intensity surface features. These contrast features are described on the basis of SEM image intensity and are not assigned to specific chemical, filler, or additive phases. Contrast-limited adaptive histogram equalization (CLAHE) was applied solely for visualization; all quantitative image analyses presented in the subsequent sections were performed using the original linear-intensity SEM images.

Figure 10 presents representative SEM micrographs of commercial silicone O-ring surfaces before hydrogen exposure and after conditioning at pressures up to 7000 psi. Across the examined fields, the surfaces remained visually continuous, with no evidence of extensive cracking, delamination, blistering, or large-scale material removal. Thus, no catastrophic surface degradation was evident within the SEM fields examined.Additional SEM micrographs of the commercial silicone O-rings under the investigated hydrogen exposure conditions are provided in the Supplementary Materials.

The unexposed specimens exhibited a relatively homogeneous silicone surface containing localized bright and dark contrast features. Following hydrogen exposure, the overall surface remained continuous, while localized differences in the distribution, morphology, and spatial organization of these contrast features became apparent among the investigated conditions.

Because elemental or spectroscopic compositional analysis was not performed, the bright and dark SEM features are not assigned to specific filler, additive, or chemical phases in the present study. The bright regions are therefore described as high-intensity surface features, while the dark regions are described as low-intensity surface features. Their contrast may arise from local topography, composition, charging behavior, or a combination of these effects. Accordingly, the SEM analysis is restricted to comparative morphology and image-intensity-based feature organization rather than chemical identification.

Importantly, these morphological changes did not increase monotonically with hydrogen pressure. Intermediate exposure conditions displayed more pronounced localized surface irregularities, whereas specimens exposed to the highest pressure retained a comparatively continuous surface without evidence of interconnected crack networks or extensive fragmentation. These observations are consistent with localized differences in the morphology and spatial organization of existing surface features rather than the formation of extensive macroscopic damage. However, because hydrogen uptake, swelling, desorption kinetics, chemical changes, and filler–polymer interactions were not directly measured, the SEM observations alone cannot establish the mechanism responsible for these morphological differences. Overall, the qualitative SEM observations indicate that the examined silicone surfaces remained visually continuous after prolonged hydrogen exposure while exhibiting localized pressure-associated morphological differences. These observations provide the qualitative basis for the quantitative image analyses presented in the following sections, where segmentation-based descriptors, spatial statistics, and topological metrics are used to characterize differences in the observed surface features and their spatial organization.

### 4.2. Automated Image Segmentation and Feature Identification

**Figure 11:**
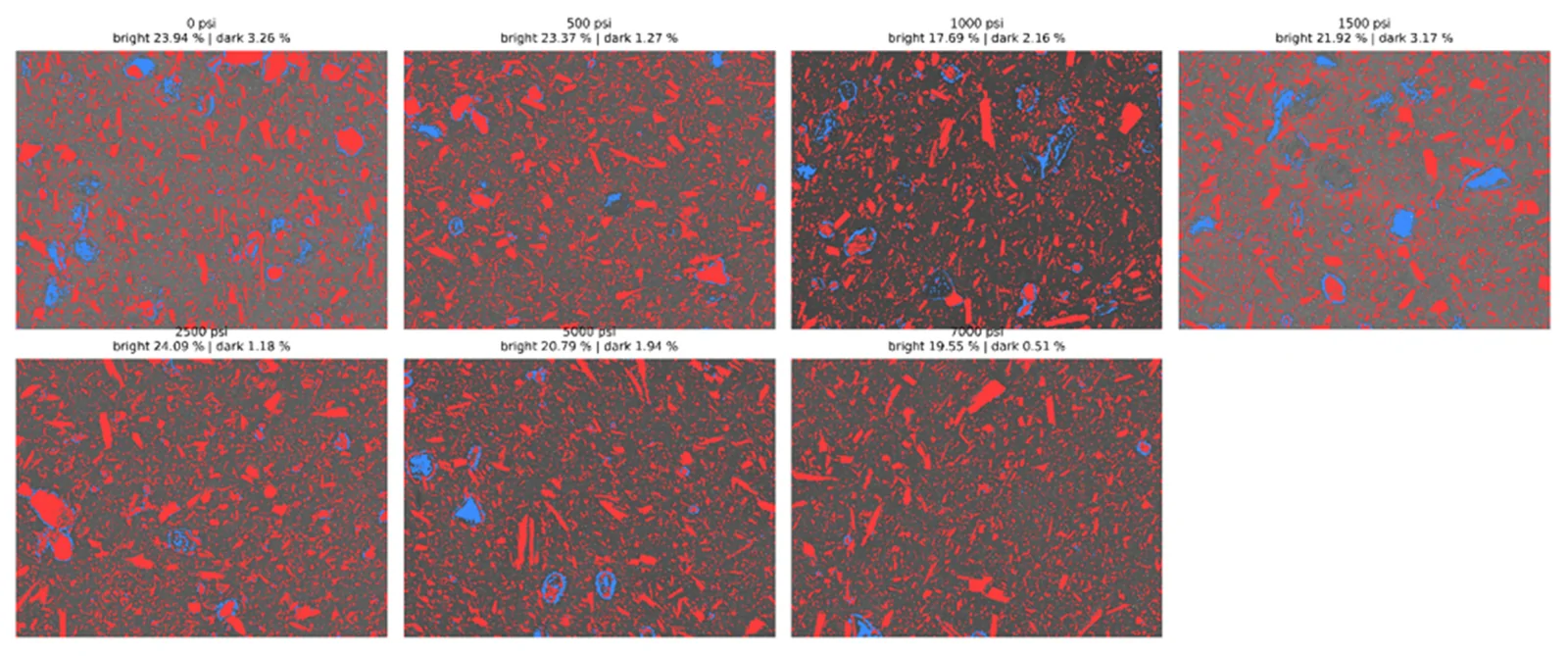
Automated segmentation overlays of representative SEM micrographs of commercial silicone O-ring surfaces following hydrogen conditioning at 0, 500, 1000, 1500, 2500, 5000, and 7000 psi. Bright high-intensity features are highlighted in red, while dark low-intensity surface features are identified in blue. The percentages above each image indicate the segmented bright and dark area fractions. These binary feature maps provide the basis for the quantitative characterization of surface morphology, feature distribution, spatial organization, and topological evolution presented in the subsequent analyses.

To enable objective comparison of surface morphology, the SEM micrographs were subjected to automated image segmentation based on grayscale contrast, separating bright high-intensity regions from dark low-intensity regions relative to the surrounding silicone matrix. These segmented regions are treated as image-defined morphological features and are not assigned to specific chemical or filler phases because compositional analysis was not performed. Representative segmentation results illustrating the identified bright and dark surface features under different hydrogen exposure conditions are presented in Figure 11.

The segmentation workflow consistently identified both bright and dark surface features across all hydrogen exposure conditions, producing comparable binary feature maps despite local variations in image contrast. The bright phase occupied approximately 17.7–24.1% of the analyzed surface, whereas the segmented dark phase accounted for only 0.5–3.3% of the total image area. Bright-feature coverage remained relatively stable throughout the investigated pressure range, whereas the dark-feature fraction exhibited comparatively larger pressure-associated fluctuations, indicating that the observed differences were concentrated in localized image-defined surface features rather than in broad changes in overall surface coverage.

Although the segmented area fractions varied only modestly, the spatial distribution and morphology of the identified features differed more noticeably among the exposure conditions. Several analyzed fields exhibited similar bright or dark area fractions despite clear differences in feature arrangement and local concentration, indicating that area fraction alone cannot fully describe the observed surface differences. Accordingly, additional quantitative descriptors of feature density, spatial organization, and topology were used to characterize the pressure-associated morphological variation. The segmentation results therefore provide the quantitative foundation for the subsequent image analyses by converting the qualitative SEM observations into reproducible binary feature maps suitable for systematic comparison across the hydrogen exposure conditions.

### 4.3. Quantitative Morphological Variation

**Figure 12:**
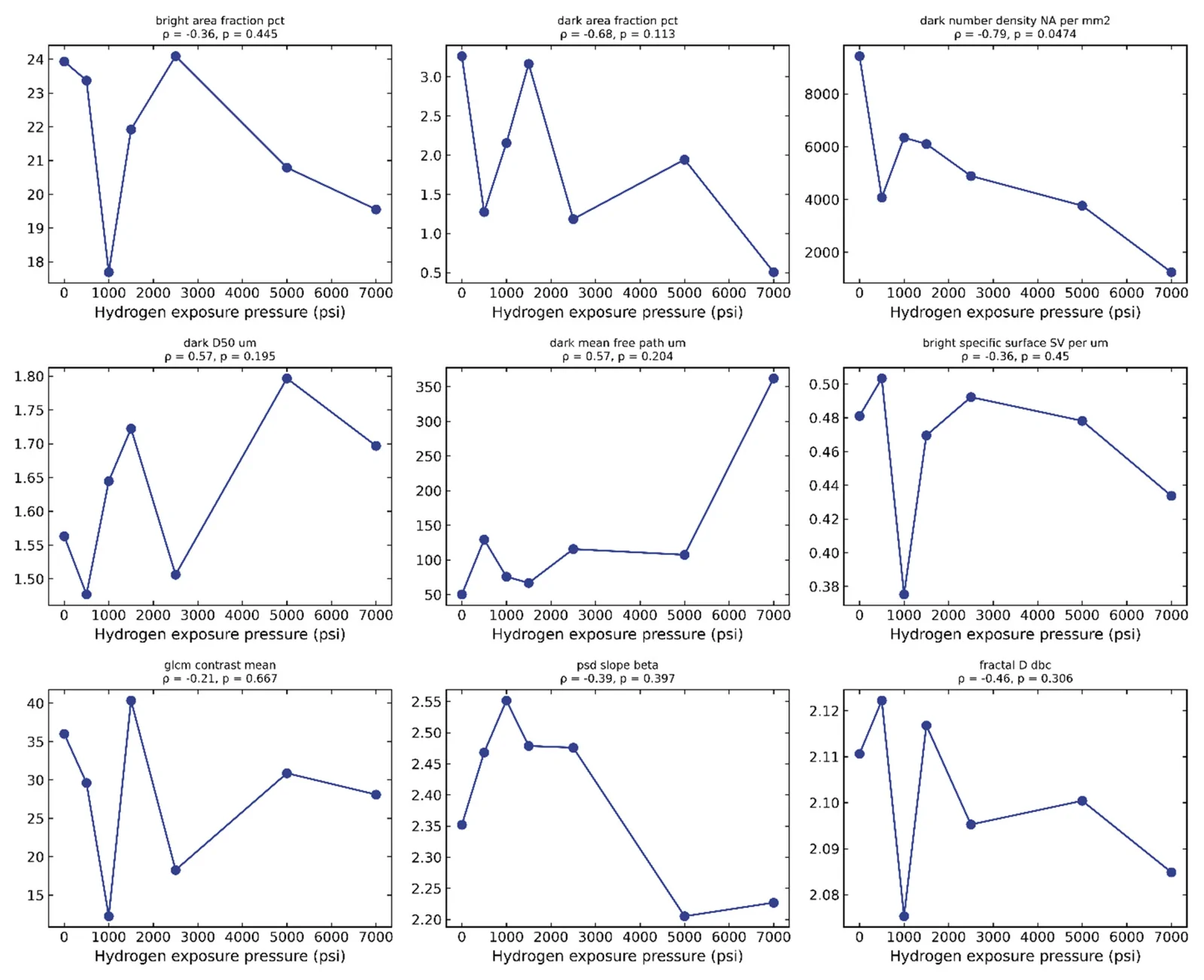
Pressure-dependent quantitative morphological descriptors extracted from segmented SEM images of silicone following hydrogen conditioning. The descriptors include bright and dark area fractions, dark feature number density, median equivalent diameter (D50), mean free path between neighboring dark features, bright-phase specific surface, GLCM contrast, power spectral density (PSD) slope, and fractal dimension. Together, these measurements quantify changes in surface coverage, feature density, geometric characteristics, texture, and surface complexity as a function of hydrogen exposure pressure. The values above each subplot represent the Spearman rank correlation coefficient (ρ) and corresponding *p* value calculated across the analyzed pressure-level fields and are presented as exploratory indicators of monotonic pressure association.

The qualitative surface morphology and segmented feature identification are presented in Figure 10 and Figure 11, respectively, providing the basis for the subsequent quantitative analysis.The quantitative descriptors extracted from the segmented SEM images provide an objective characterization of pressure-associated morphological variation across the analyzed conditions (Figure 12). Whereas the qualitative observations in Figure 10 illustrate the overall morphological patterns and Figure 11 provides reproducible feature identification, the descriptors presented here quantify differences in surface coverage, feature morphology, spatial organization, and texture, enabling systematic comparison among the hydrogen exposure conditions.

The segmented bright features occupied approximately 18–24% of the analyzed surface, whereas the dark features consistently represented less than 3.5% of the total image area. Although both descriptors exhibited moderate variation among the pressure conditions, neither showed a consistent monotonic trend. The analyzed fields therefore showed comparatively limited pressure-associated variation in overall bright- and dark-feature surface coverage.

More pronounced pressure-associated variation was observed in descriptors characterizing the dark surface features. Dark-feature number density exhibited an overall decrease with increasing hydrogen pressure, with Spearman analysis across the analyzed pressure-level fields yielding ρ = −0.79 and *p* = 0.0474. Given the representative-field nature of the SEM analysis, this result is interpreted as an exploratory monotonic association rather than as a population-level statistical effect. In contrast, the median equivalent diameter (D50) and mean free path tended to be higher at several elevated-pressure conditions, but their corresponding pressure associations were weaker (ρ = 0.57, *p* = 0.195 for D50; ρ = 0.57, *p* = 0.204 for mean free path). Taken together, the analyzed micrographs suggest a tendency toward fewer, and at some elevated-pressure conditions, larger and more widely separated dark surface features; however, these observations should be interpreted as morphological tendencies within the analyzed fields rather than statistically established material-wide trends.

Overall, the quantitative image descriptors indicate that the analyzed silicone surfaces exhibited greater pressure-associated variation in localized morphological features than in global surface characteristics. These observations complement the qualitative SEM findings and provide the basis for evaluating feature-level spatial organization using the spatial statistical analyses presented in the following section.

### 4.4. Spatial Organization of Pressure-Associated Surface Features

While the quantitative descriptors presented in Figure 12 characterize feature density, size, and surface coverage, Ripley’s L-function was used to examine how the segmented features were spatially organized within each analyzed SEM field. In the revised analysis, each pressure condition and feature class was compared with its own CSR reference generated using the corresponding observed feature count and observation window. This avoids applying a common CSR envelope to patterns having substantially different feature densities.

The bright-feature patterns exhibited comparatively small and condition-dependent departures from CSR. Several conditions showed localized departures above or below their corresponding CSR bounds over limited distance ranges, whereas others showed predominantly spatial inhibition over portions of the evaluated range. No consistent monotonic progression in bright-feature spatial organization was observed with increasing hydrogen pressure. The bright-feature results are therefore interpreted as field-level differences in spatial arrangement rather than evidence of a systematic pressure-driven transition.

The dark-feature patterns showed a markedly different response. The observed (L(r)-r) curves exceeded their corresponding upper CSR bounds over the evaluated distance range for both the unexposed and hydrogen-exposed conditions, demonstrating that clustering of dark features was already present in the unexposed surface. The magnitude and length-scale dependence of the clustering nevertheless differed among the investigated pressure conditions. In particular, several exposed fields exhibited larger departures from their corresponding CSR references than the unexposed field, but the response was non-monotonic with pressure.

Accordingly, departure from CSR alone is not interpreted as evidence that hydrogen exposure generated clustering. Instead, the Ripley analysis demonstrates that dark-feature clustering is an existing characteristic of the analyzed surface morphology, while the relative magnitude and scale of spatial organization vary among the investigated conditions. Given the field-level nature of the SEM sampling, these differences are interpreted as exploratory morphological observations rather than specimen-level statistical evidence of an exposure-induced spatial transition.

### 4.5. Topological Characterization of Pressure-Associated Surface Morphology

**Figure 13:**
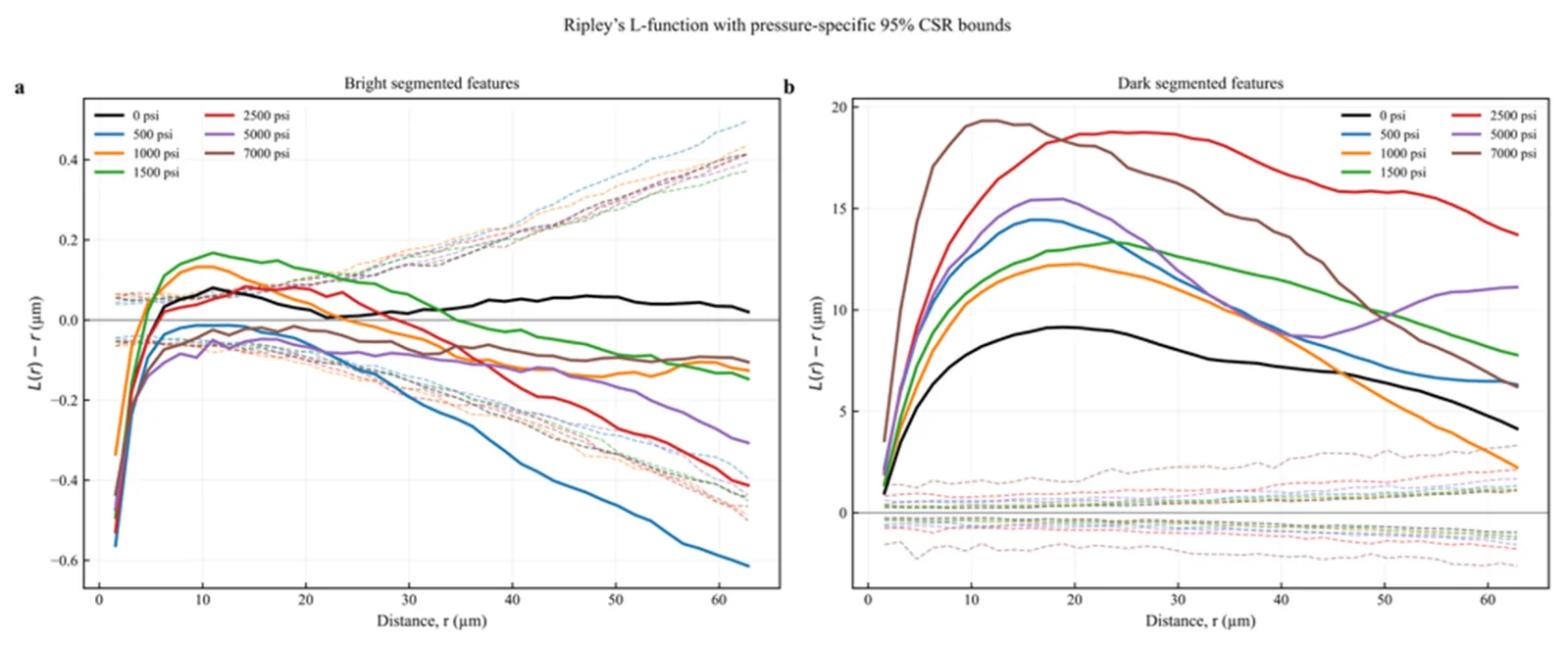
Ripley’s L-function analysis of the spatial organization of segmented (**a**) bright and (**b**) dark surface features following hydrogen conditioning. Solid curves represent the observed (L(r)-r) response for each exposure condition. The corresponding color-matched dashed curves represent the pressure- and feature-specific pointwise 95% CSR bounds obtained from 199 Monte Carlo realizations using the same number of points and the same rectangular observation window as the corresponding observed pattern. Values above the corresponding upper CSR bound indicate greater clustering than expected under CSR at that distance, whereas values below the lower bound indicate spatial inhibition. The CSR comparison characterizes each individual field-level spatial pattern and is not, by itself, interpreted as evidence of a hydrogen-exposure effect.

**Figure 14:**
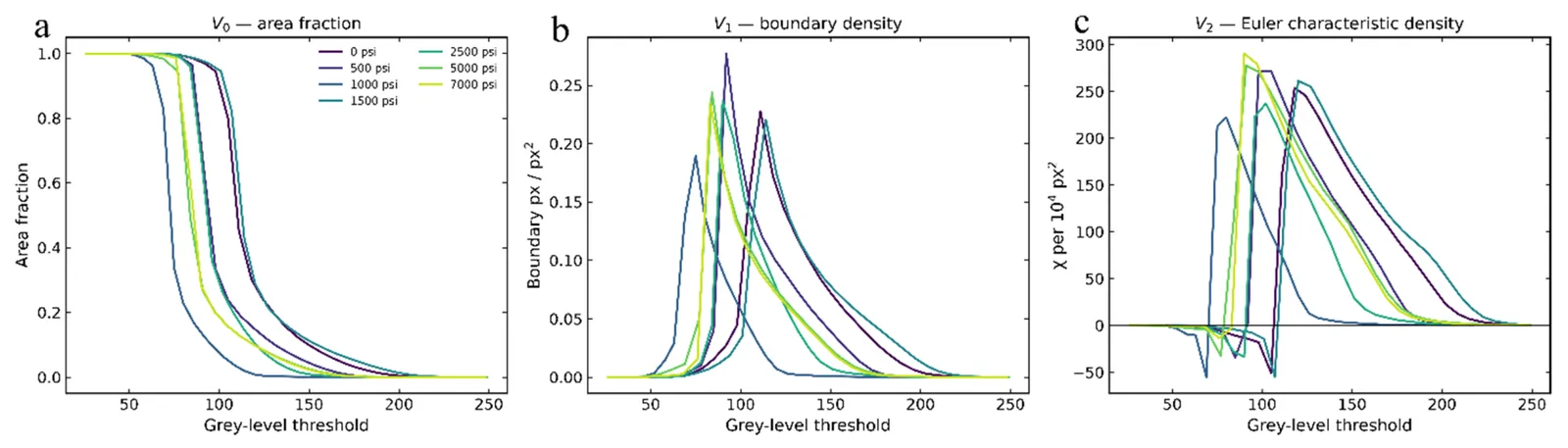
Minkowski functional analysis describing the global topological evolution of silicone surface morphology after hydrogen exposure. (**a**) Area fraction (V_0_), (**b**) boundary density (V_1_), and (**c**) Euler characteristic density (V_2_) evaluated over the full grayscale threshold range. Differences in transition position, boundary density, and Euler characteristic describe variations in threshold-dependent image morphology and topology among the analyzed conditions. The Euler characteristic represents the number of connected foreground components minus the number of enclosed holes and is not interpreted as a direct measure of physical polymer-network connectivity.

While Figure 13 describes the spatial organization of segmented surface features, the Minkowski functional analysis presented in Figure 14 evaluates the evolution of the overall surface topology across the complete grayscale threshold range. Unlike object-based descriptors or spatial statistics, Minkowski functionals characterize changes in area fraction, interface complexity, and threshold-dependent topology, thereby providing a complementary description of pressure-associated image morphology.

The area fraction functional (V_0_, Figure 14a) exhibited a similar sigmoidal transition for all exposure conditions, indicating that the overall morphological framework remained comparable throughout the investigated pressure range. However, modest shifts in the transition threshold were observed among the exposure conditions, reflecting pressure-dependent changes in grayscale intensity distribution associated with subtle surface morphological evolution.

The boundary density functional (V_1_, Figure 14b) showed a distinct maximum for each condition, corresponding to the threshold at which the greatest interface density was generated during segmentation. Although the overall curve profiles remained similar, modest variations in peak position and distribution width indicate small changes in interface complexity following hydrogen exposure. In particular, the broader distributions observed at several higher exposure pressures are consistent with the gradual evolution of surface interfaces rather than the abrupt formation of new morphological boundaries.

The Euler characteristic density (V_2_, Figure 14c) showed differences among the investigated exposure conditions across the grayscale-threshold range. In a two-dimensional thresholded image, the Euler characteristic corresponds to the number of connected foreground components minus the number of enclosed holes. Positive values therefore indicate that connected components outnumber holes, whereas negative values indicate a greater contribution from enclosed holes. Differences in the position and magnitude of the (V_2_) curves consequently describe changes in the threshold-dependent balance between segmented components and holes. They do not demonstrate preservation or disruption of a physically connected polymer network.

Overall, the Minkowski functional analysis complements the segmentation and spatial analyses by identifying comparatively subtle differences in grayscale distribution, interface density, and threshold-dependent image topology among the analyzed fields. These descriptors are therefore interpreted as image-based morphological measures and not as direct evidence of polymer-network connectivity or a specific hydrogen-induced damage mechanism.

## 5. Limitations of the Present Study

The present study focused on the post-exposure mechanical and surface characterization of commercially available silicone O-rings following prolonged hydrogen exposure under room-temperature conditions. The investigation was limited to a single commercial silicone formulation and a single exposure duration, while complementary characterization techniques such as hydrogen permeation, swelling measurements, spectroscopic analysis, and long-term environmental aging were beyond the scope of the present work.

FTIR spectroscopy and thermal or dynamic-mechanical characterization were not included in the present experimental program. Consequently, the study does not directly evaluate changes in chemical bonding, additive–polymer interactions, glass-transition behavior, or molecular chain mobility following hydrogen exposure. Any discussion of plasticization, altered chain mobility, or other physicochemical mechanisms is therefore treated only as a possible interpretation of the observed macroscopic mechanical response rather than as an experimentally confirmed mechanism. Future work should combine the present tensile framework with FTIR and DSC or DMA measurements before and after hydrogen exposure to determine whether the observed mechanical changes are accompanied by measurable chemical or viscoelastic transitions.

The quantitative SEM analysis was based on representative surface fields and was intended to characterize pressure-associated morphological patterns rather than to establish population-level surface variability. Because elastomer surfaces can exhibit local spatial heterogeneity, the analyzed fields may not capture the complete distribution of morphological features across each O-ring surface. Accordingly, SEM-derived correlations and spatial descriptors are interpreted as supportive, field-level evidence and should be validated in future studies using broader specimen-level and multi-field sampling.

The mechanical dataset includes an unexposed 0 psi reference, enabling direct comparison of the tensile response with the post-exposure conditions. Nevertheless, the unexposed group exhibited substantial specimen-to-specimen variability in several mechanical quantities, and the number of replicates differed among conditions. The results should therefore be interpreted using both group-level statistics and specimen-level variability. In addition, the study evaluated one commercial silicone formulation, one exposure duration, and discrete pressure conditions; consequently, the observed non-monotonic response should not be generalized as a universal pressure–response relationship for silicone elastomers.

Although the investigated pressure range of 0–7000 psi encompasses conditions relevant to many high-pressure hydrogen sealing applications, the discrete pressure levels evaluated in the present study do not allow the partial recovery observed at 5000 psi to be interpreted as a recurring turning point or characteristic pressure threshold. Future investigations should therefore extend the analysis to broader and more closely spaced pressure ranges, including pressures above 7000 psi, to determine whether additional non-monotonic transitions occur. The present study also considered fixed-pressure exposure conditions followed by a single controlled depressurization and did not evaluate repeated pressure fluctuations or pressurization–depressurization cycling. Future work should therefore examine pressure-history effects, including repeated cycling, pressure-change amplitude, pressurization and depressurization rates, and dwell time, because these factors may influence hydrogen transport and the resulting mechanical response of elastomeric seals.

All hydrogen exposures and post-exposure mechanical evaluations in the present study were conducted at room temperature. Although silicone elastomers are attractive for sealing applications because of their broad thermal flexibility, their mechanical and viscoelastic behavior is strongly temperature dependent [27]. The present results should therefore not be directly extrapolated to sub-ambient, cryogenic, or elevated-temperature hydrogen-service conditions, where changes in polymer-chain mobility, thermal contraction, stiffness, stress relaxation, and elastic recovery may alter sealing behavior. Future studies should incorporate coupled pressure–temperature test matrices together with temperature-dependent characterization methods such as dynamic mechanical analysis, low-temperature tensile testing, stress–relaxation measurements, and seal-level compression or leakage evaluation. Such measurements would provide a more complete understanding of temperature-dependent stiffness, damping, glass-transition behavior, recovery, and sealing performance. Constitutive modeling, finite-element analysis, and molecular-scale simulations could further complement these experiments by linking the measured macroscopic response to temperature-dependent polymer-network behavior and hydrogen transport.

In addition, silicone formulations containing different filler types, filler loadings, crosslinking densities, and curing chemistries should be systematically investigated to establish composition–structure–property relationships governing hydrogen compatibility. Such integrated mechanical, viscoelastic, physicochemical, computational, and microstructural characterization across broader material formulations and service conditions would provide a more comprehensive understanding of hydrogen–silicone interactions and support the development and qualification of more reliable elastomeric sealing materials for hydrogen infrastructure.

## 6. Conclusions

The addition of an unexposed 0 psi mechanical reference enabled direct assessment of the tensile response relative to the initial material condition. The unexposed group exhibited a mean tensile strength of 697 ± 208 psi, ultimate elongation of 98.6 ± 36.0%, tensile toughness of 380 ± 233 psi, and structural loading work of 17.8 ± 10.9 lbf·in. Several hydrogen-exposed groups exhibited greater deformation capacity and loading work to failure than the unexposed reference, with mean elongation reaching 188.1% at 500 psi and 182.3% at 5000 psi. The response was non-monotonic rather than progressively increasing or decreasing with pressure, and the 7000 psi group showed lower elongation and energy-related properties than selected lower-pressure exposed groups.

Welch ANOVA identified significant overall group effects for tensile strength (*p* = 0.0073), ultimate elongation (*p* = 0.0068), tensile toughness (*p* = 0.0065), and structural loading work (*p* = 0.0065). Pairwise analysis showed that the unexposed group had significantly lower elongation, toughness, and structural loading work than the 500, 1000, and 5000 psi groups, while selected differences were also observed among the hydrogen-exposed conditions. These findings demonstrate an exposure-associated change in deformability and failure response but do not, by themselves, establish the molecular mechanism responsible for the observed behavior.

Multivariate analysis using the reduced five-descriptor feature set further showed that the mechanical response remained distributed across overlapping specimen-level response space rather than forming uniquely separated pressure-defined states. The first two principal components accounted for approximately 79.3% of the total variance (PC1: 43.4%; PC2: 35.9%). The corresponding loading structure showed that PC1 primarily contrasted tensile strength and ultimate elongation with stiffness-related descriptors, whereas all five selected descriptors contributed positively to PC2. The unexposed group exhibited comparatively large specimen-to-specimen variability, and the hydrogen-exposed conditions remained substantially overlapping in the PCA representation. These analyses are therefore interpreted as descriptive summaries of coordinated mechanical variation rather than as evidence of mechanical equivalence or discrete pressure-specific material states.

SEM combined with automated segmentation, spatial statistics, and Minkowski functional analysis revealed differences primarily in localized surface morphology and spatial organization, while no evidence of catastrophic surface degradation was observed in the analyzed fields. Dark surface features exhibited clustering in both the unexposed and hydrogen-exposed conditions, demonstrating that departure from complete spatial randomness was not unique to hydrogen exposure. The magnitude and length-scale dependence of this clustering varied among the investigated conditions, while the Minkowski analysis showed comparatively subtle differences in threshold-dependent image topology. These SEM results are therefore interpreted as exploratory field-level morphological observations rather than evidence of a specimen-level exposure-induced spatial transition or direct changes in polymer-network connectivity.

Overall, the revised dataset demonstrates that prolonged hydrogen exposure was associated with a non-monotonic mechanical response in the investigated commercial silicone O-rings. Several exposed groups exhibited increased deformation capacity and mechanical work to failure relative to the unexposed reference, whereas the highest investigated pressure showed reduced values relative to selected lower-pressure exposed groups. The tensile results do not establish whether these changes arise from hydrogen-induced plasticization, changes in polymer-chain mobility, microstructural damage, or other physicochemical mechanisms. Accordingly, the study provides comparative post-exposure mechanical and surface-response data rather than the direct validation of sealing performance, leakage resistance, or system-level hydrogen compatibility.

## Data Availability

The data supporting the findings of this study are available from the corresponding author upon reasonable request.

## Supplementary Materials

The following are available online at https://www.mdpi.com/article/10.3390/polym18212733/s1, Additional scanning electron microscopy (SEM) images of commercial silicone O-rings before and after high-pressure hydrogen exposure are provided in the supplementary files accompanying this article.
