# Supplementary material for: Hydrogen Compatibility of Commercial Silicone Elastomer Seals: Multiscale Mechanical and Surface Characterization Following High-Pressure Hydrogen Exposure
Source: Polymers (Basel). 2026 Nov 3;18(21):2733. doi: 10.3390/polym18212733 (PMC13766088; doi:10.3390/polym18212733)

1. 0 PSI

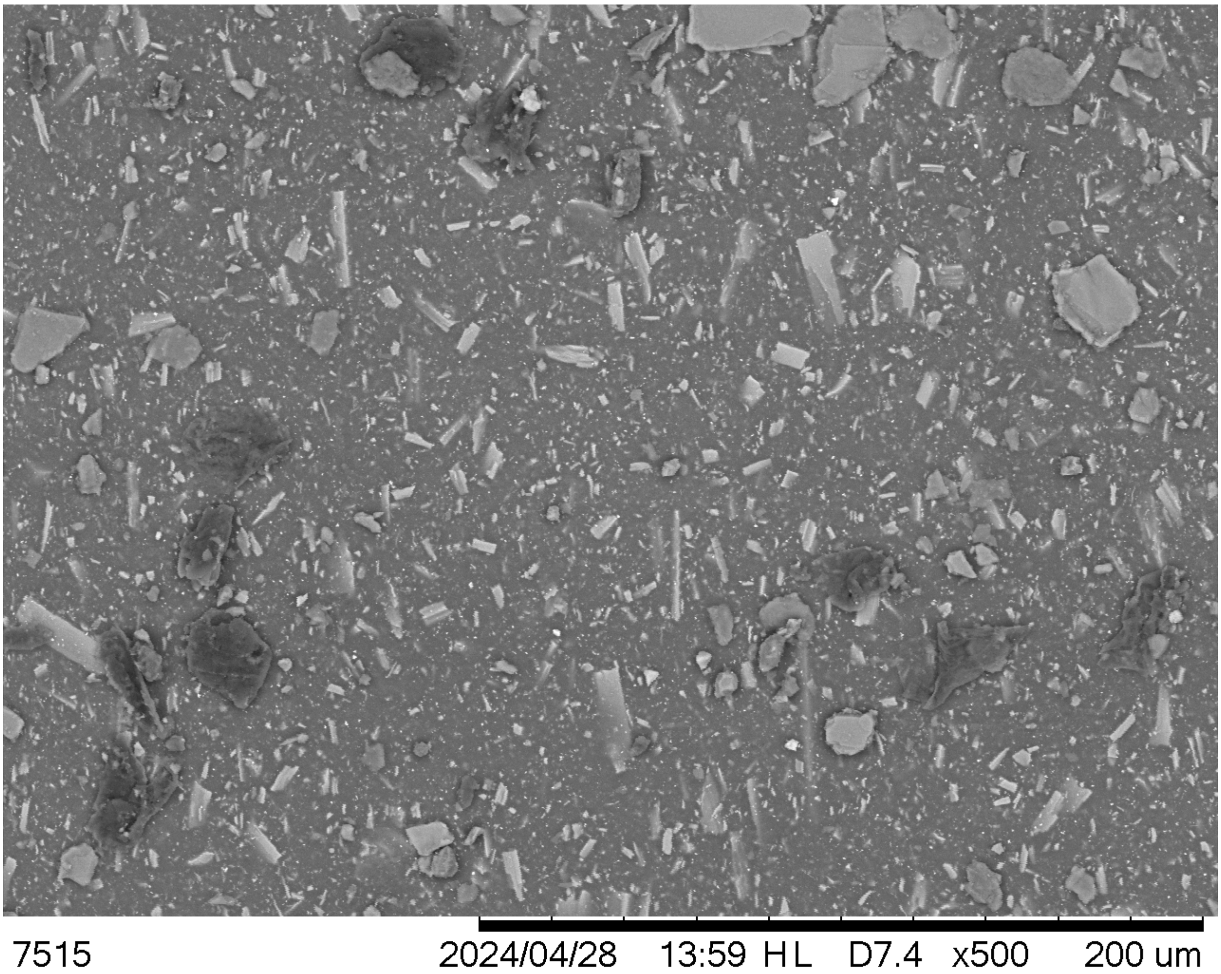

2. 500 PSI

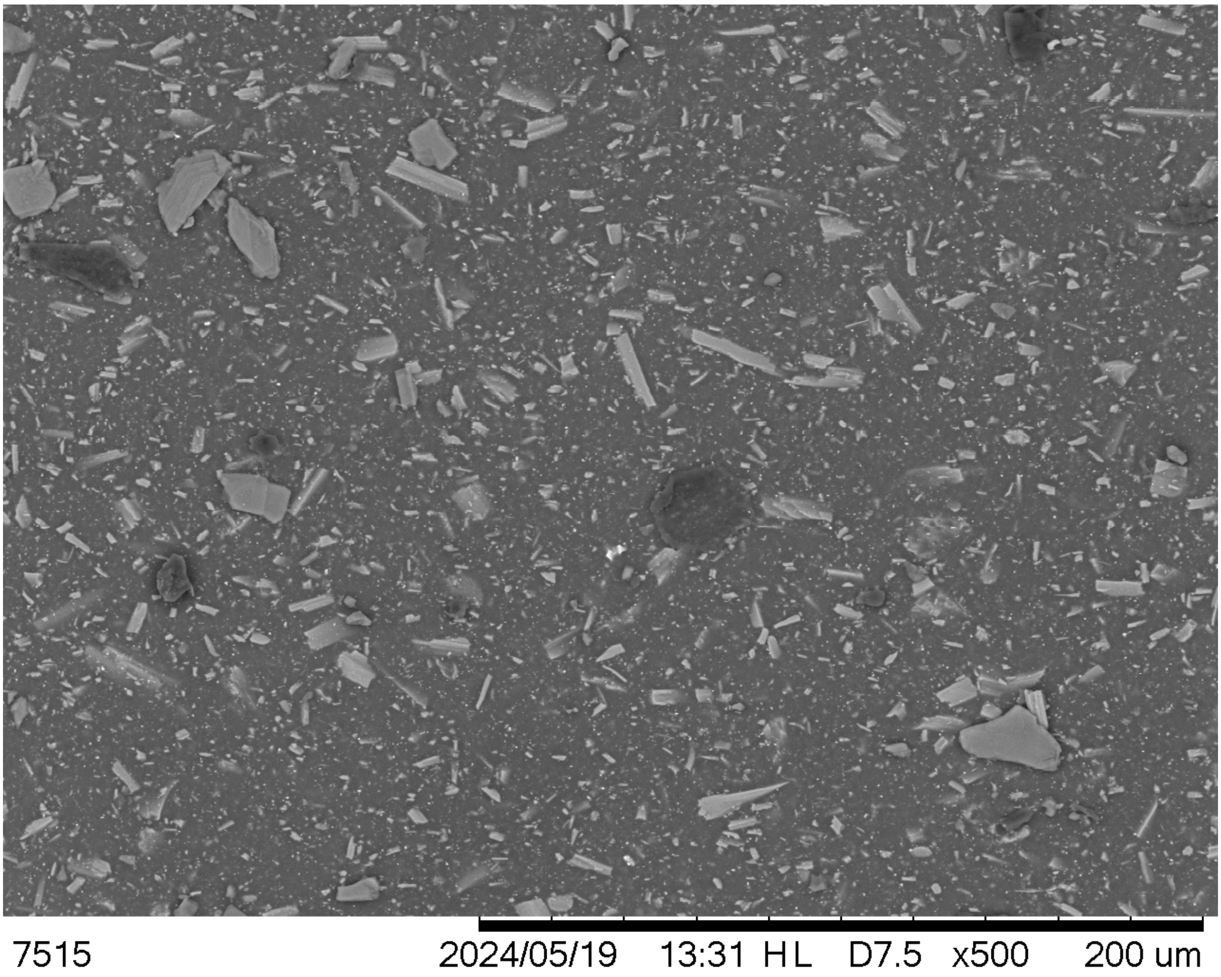

3. 1000 PSI

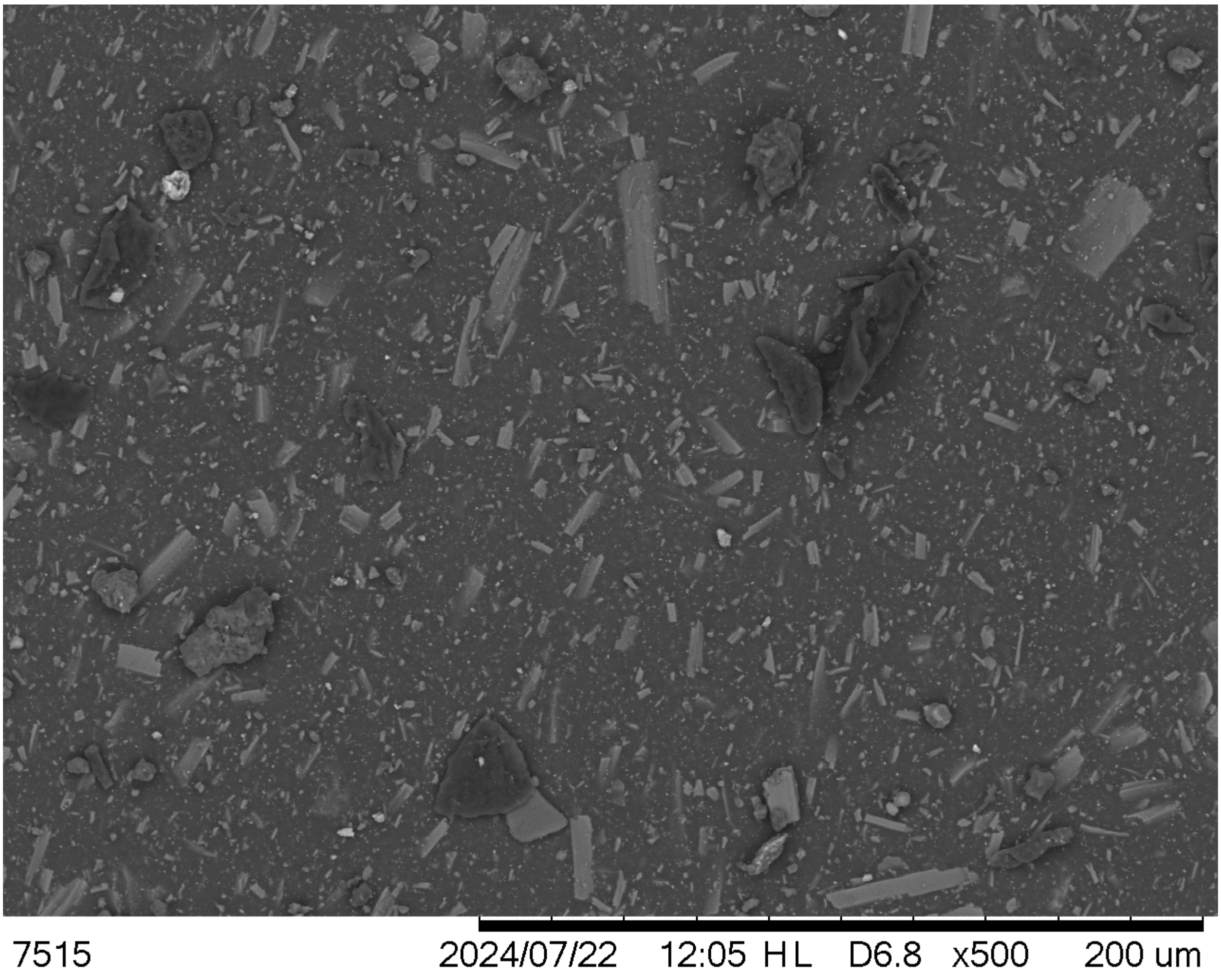

4. 1500 PSI

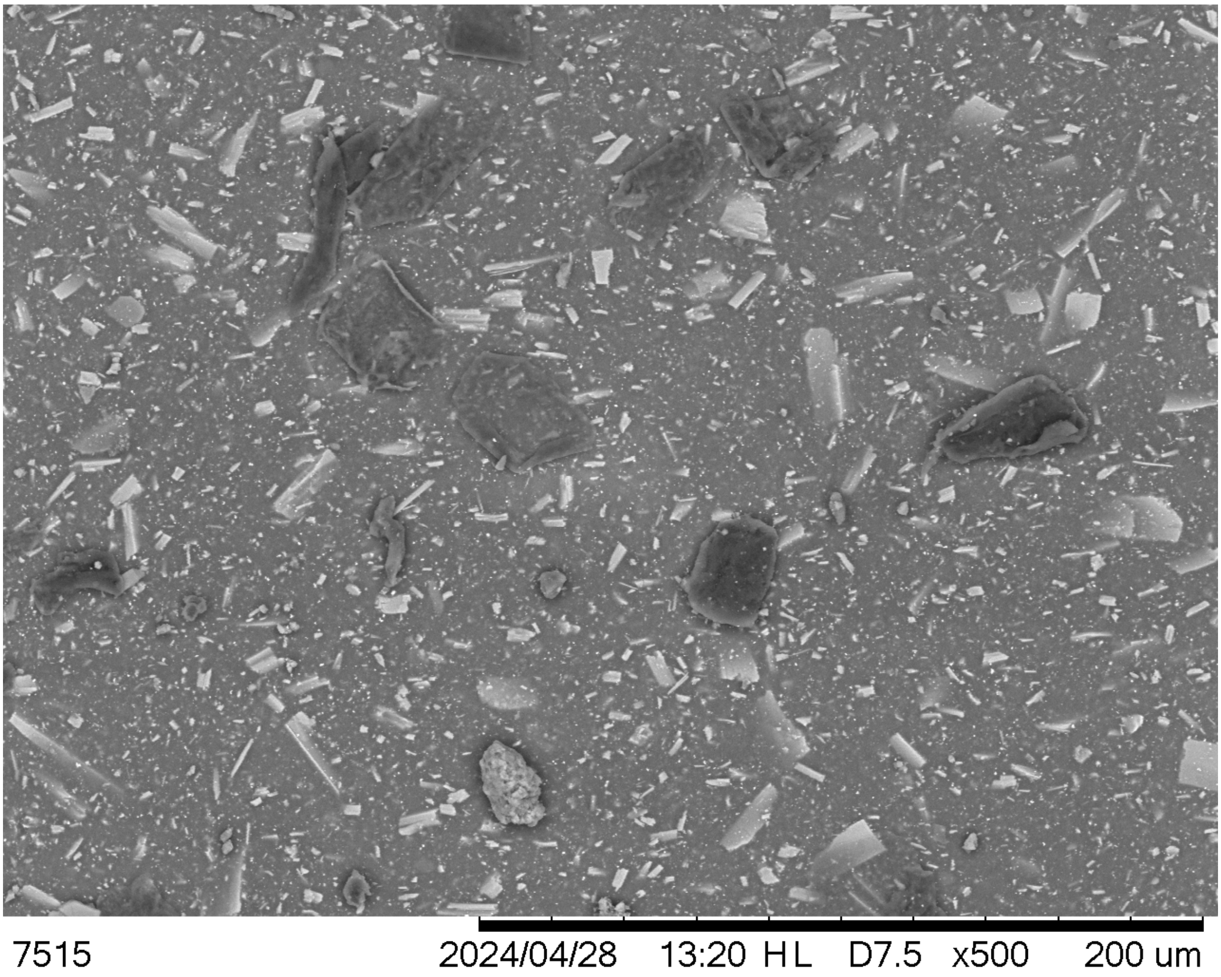

4. 2500 PSI

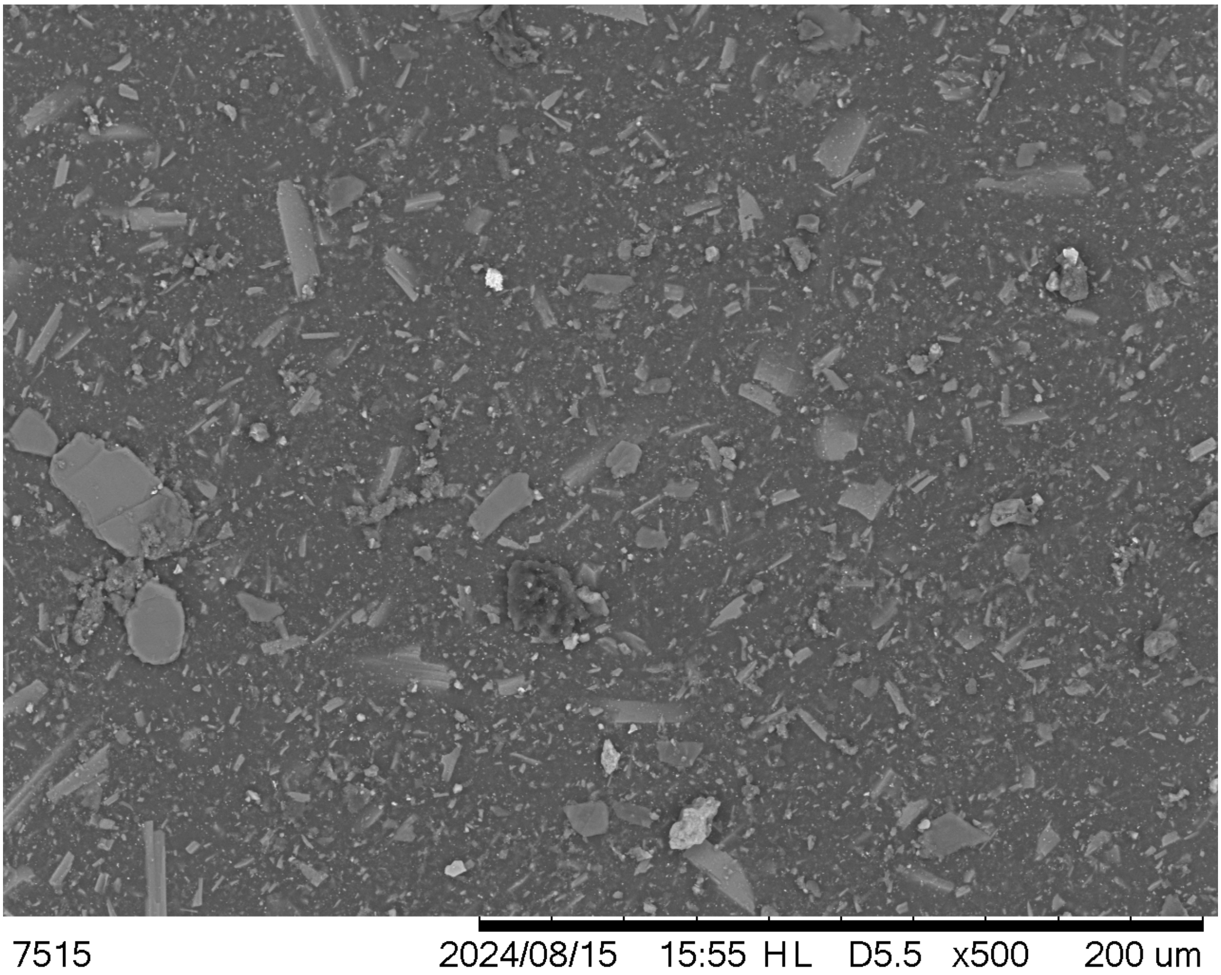

5. 5000 PSI

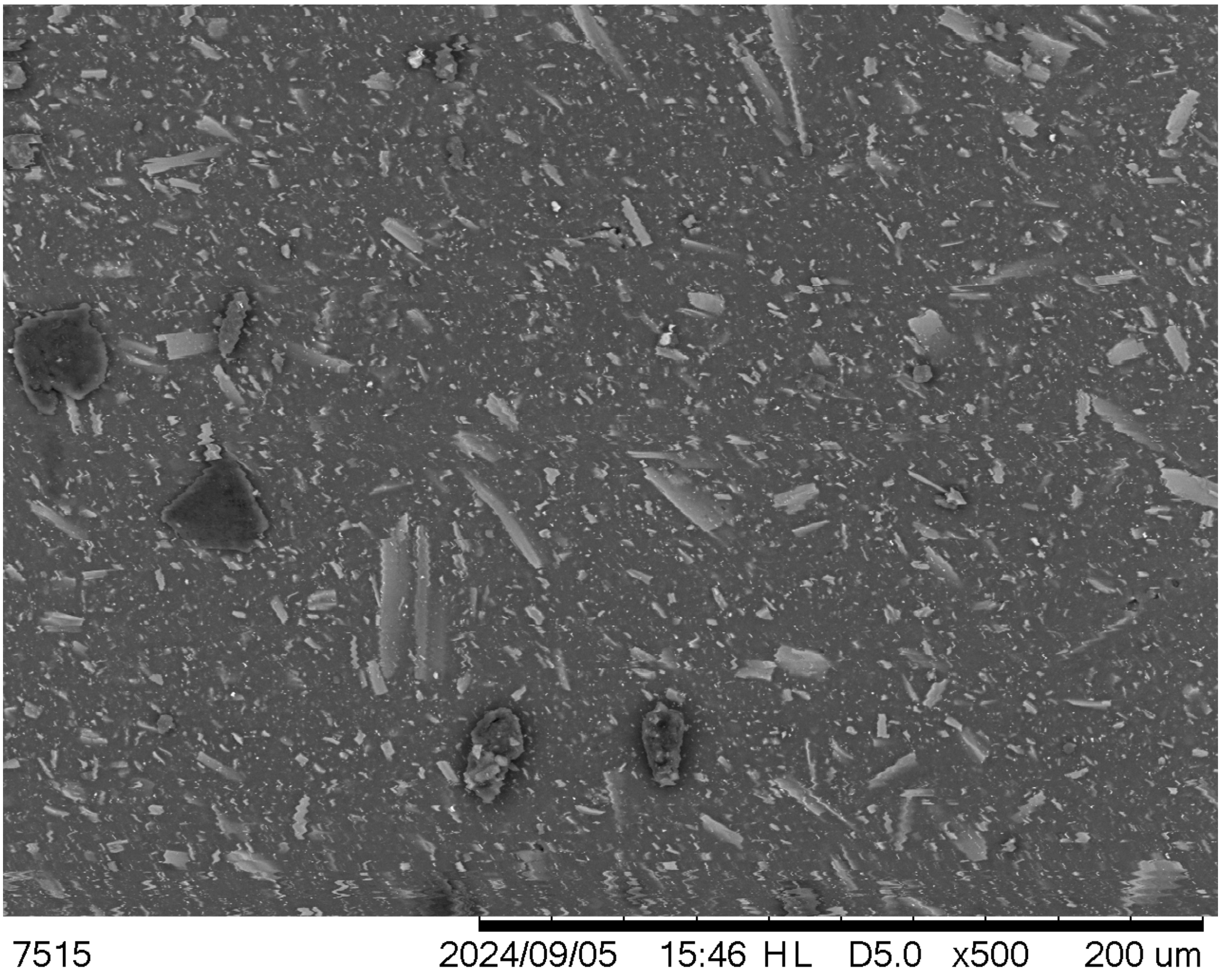

6. 7000 PSI

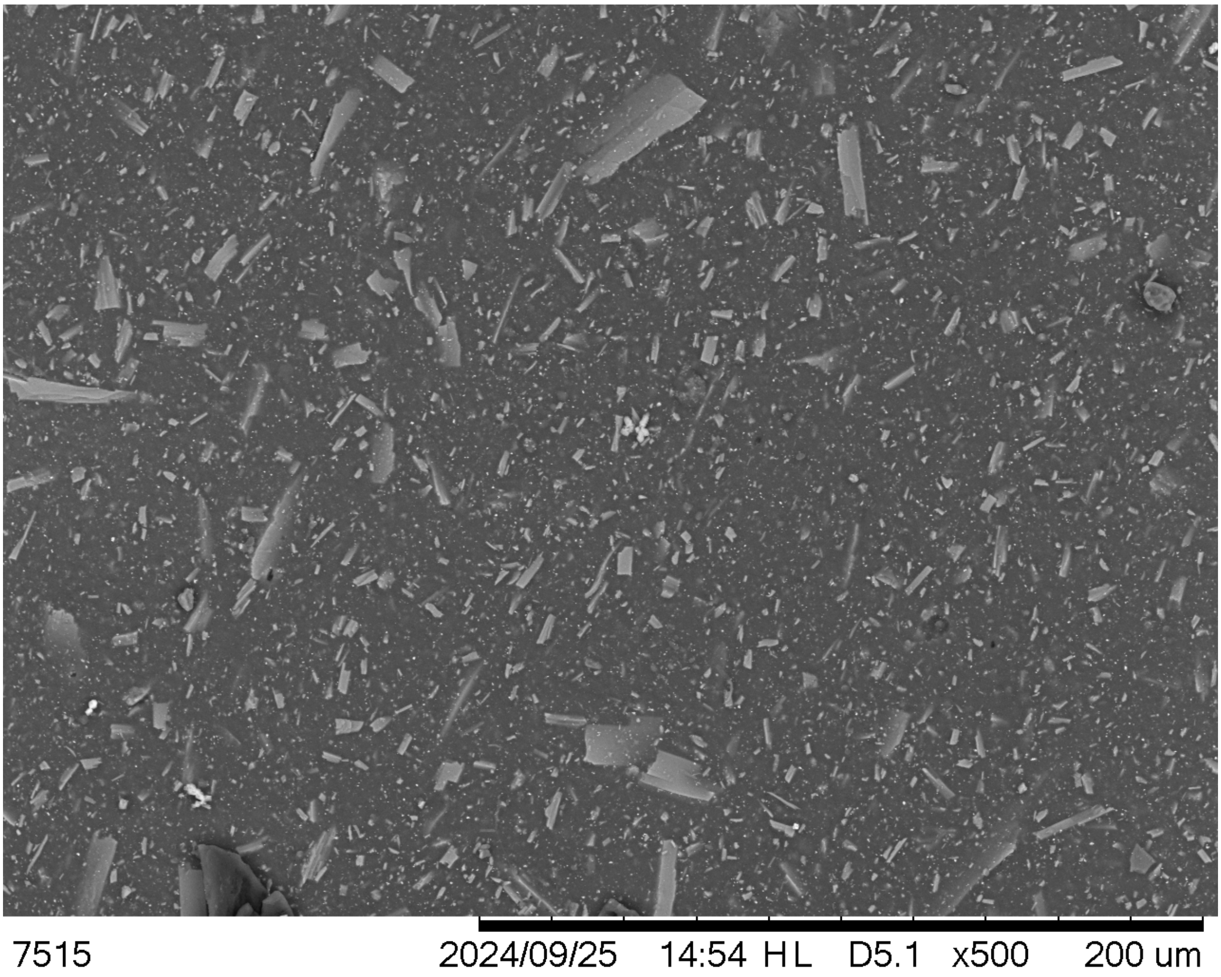
7515
2024/09/25
14:54 HL
D5.1
x500
200 um

7 0 PSI raw

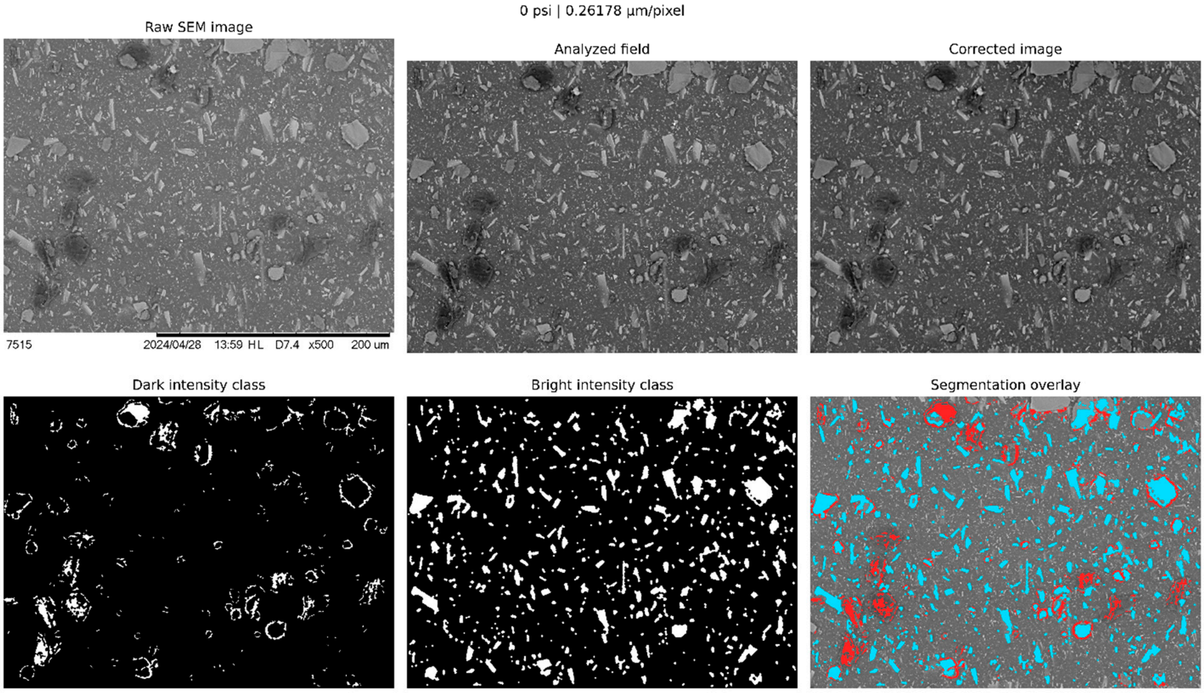

8 500 PSI raw

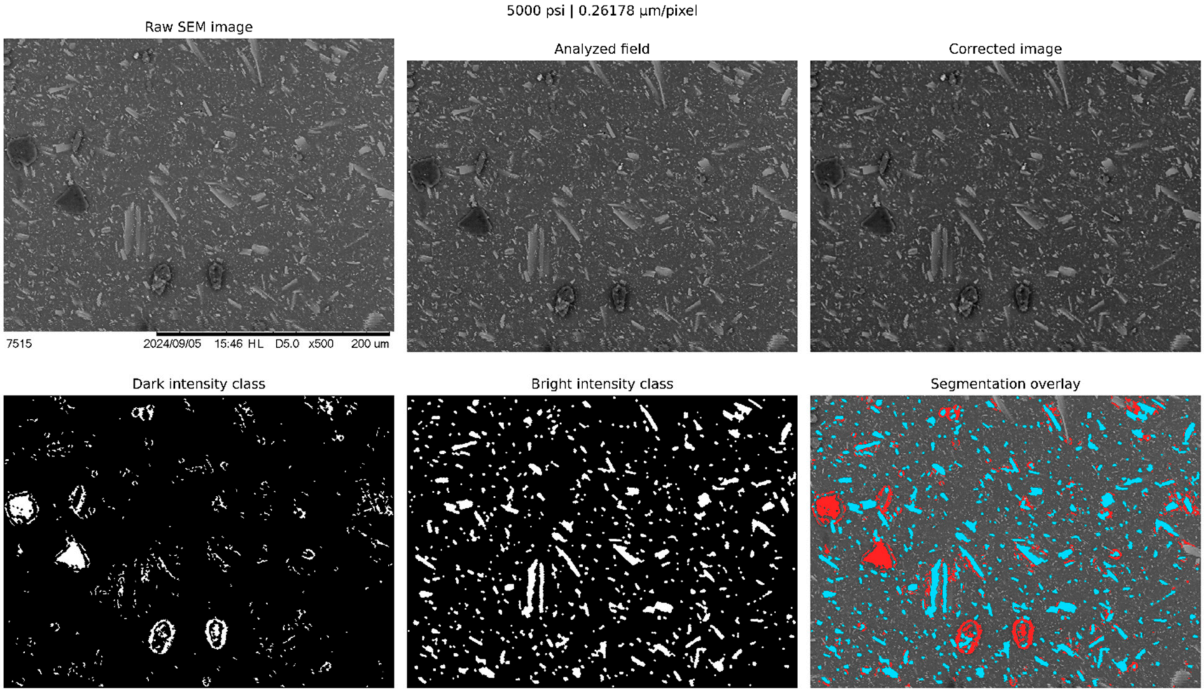

9 1000 PSI raw

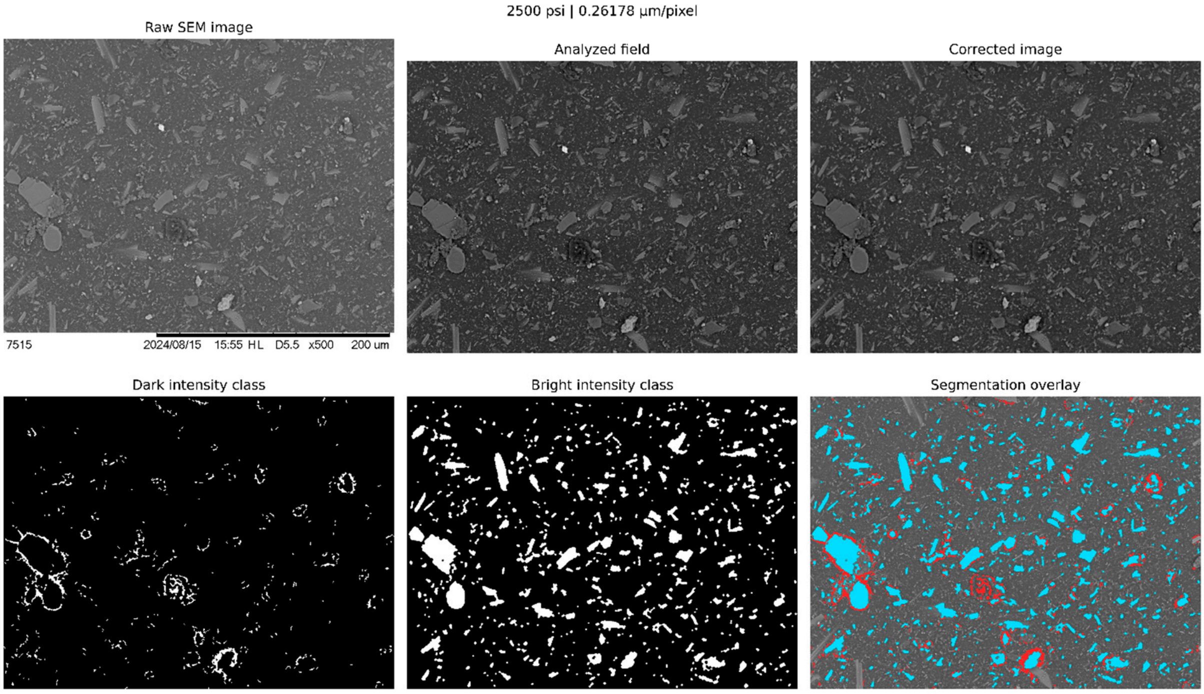

10 1500 PSI raw

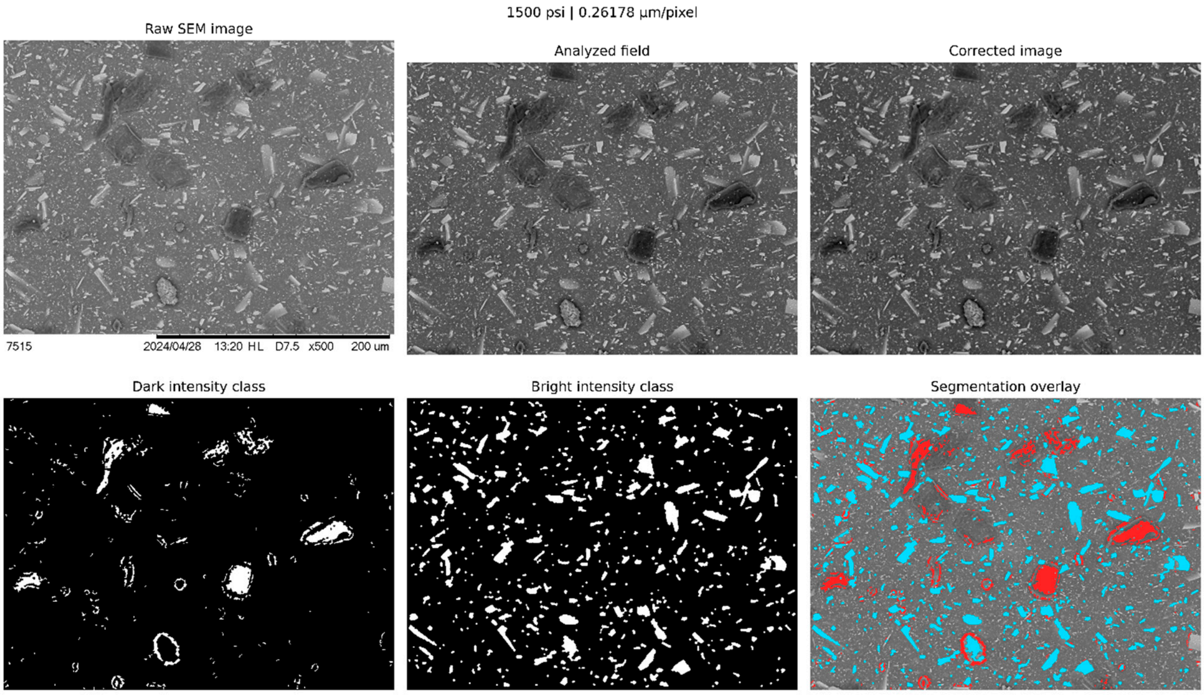

11 2500 psi raw

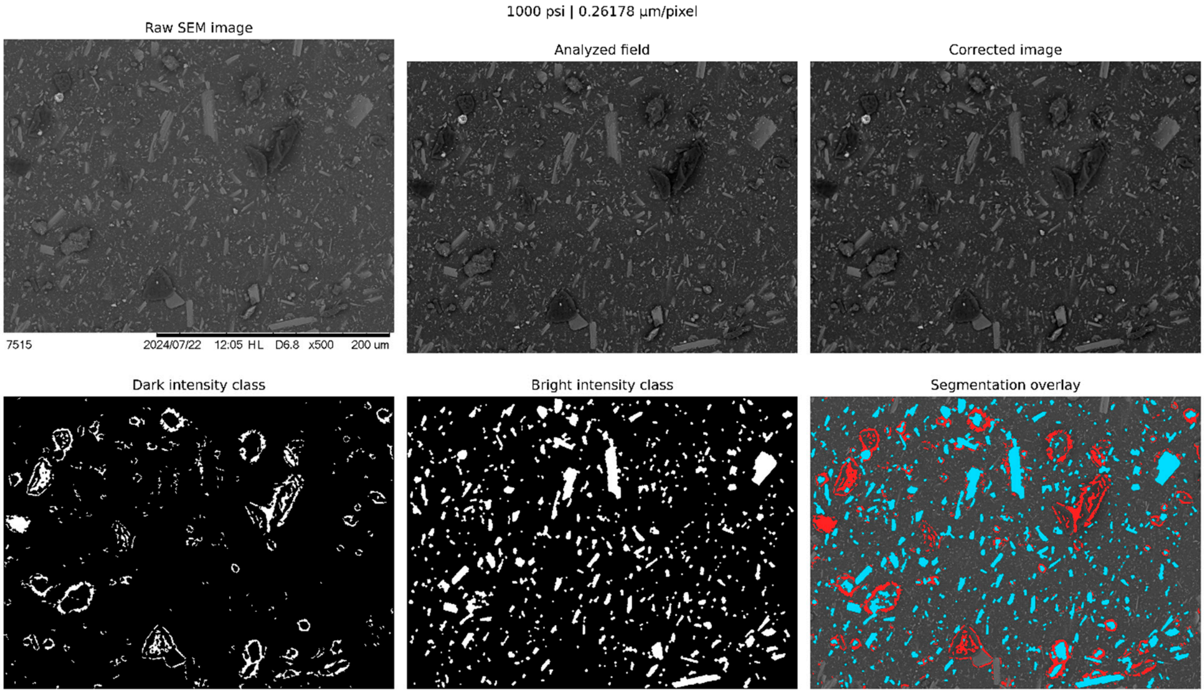

12 5000 PSI raw

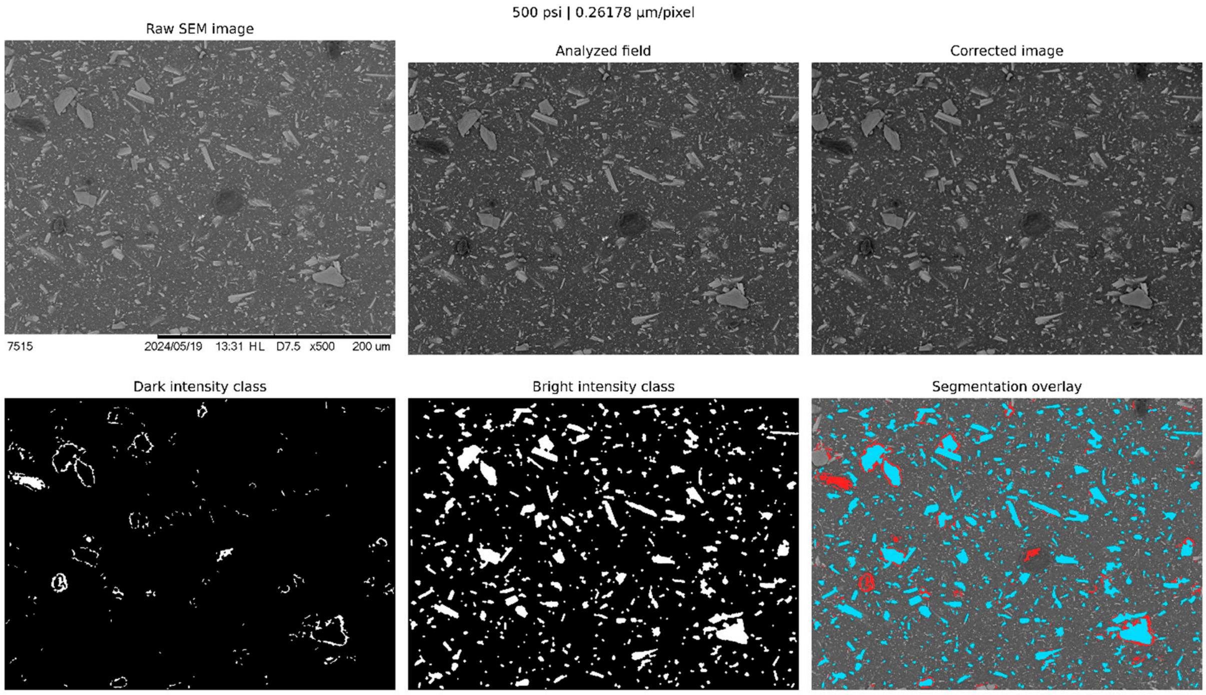

13 7000 PSI raw

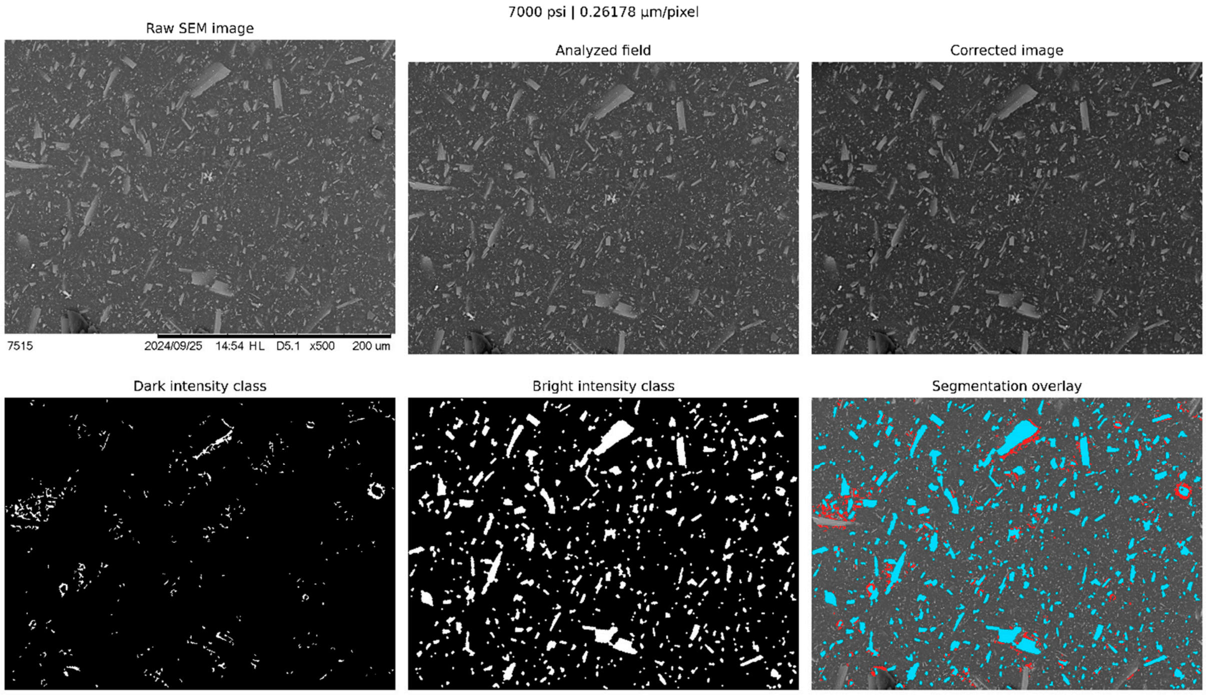

Supplement: Supplementary file 1 [file polymers-18-02733-s001.zip › polymers-4647516-supplementary.pdf]
